# Metabolome and transcriptome analysis reveals the molecular profiles underlying the ginseng response to rusty root symptoms

**DOI:** 10.1186/s12870-021-03001-w

**Published:** 2021-05-13

**Authors:** Xingbo Bian, Yan Zhao, Shengyuan Xiao, He Yang, Yongzhong Han, Lianxue Zhang

**Affiliations:** 1 College of Chinese Medicinal Materials, Jilin Agricultural University, Changchun, 130118 Jilin Province China; 2 State Local Joint Engineering Research Center of Ginseng Breeding and Application, Changchun, China; 3 Jilin Provincial Ginseng and Pilose Antler Office, Changchun, China

**Keywords:** *Panax ginseng*, Rusty root, Metabolome, Transcriptome, Environmental stress

## Abstract

**Background:**

Ginseng rusty root symptoms (GRS) is one of the primary diseases of ginseng. This disease leads to a severe decline in the quality of ginseng. It has been shown that the occurrence of GRS is associated with soil environmental degradation, which may involve changes in soil microbiology and physicochemical properties.

**Results:**

In this study, GRS and healthy ginseng (HG) samples were used as experimental materials for comparative analysis of transcriptome and metabolome. Compared with those in HG samples, 949 metabolites and 9451 genes were significantly changed at the metabolic and transcriptional levels in diseased samples. The diseased tissues’ metabolic patterns changed, and the accumulation of various organic acids, alkaloids, alcohols and phenols in diseased tissues increased significantly. There were significant differences in the expression of genes involved in plant hormone signal transduction, phenylpropanoid biosynthesis, the peroxidase pathway, and the plant-pathogen interaction pathway.

**Conclusion:**

The current study involved a comparative metabolome and transcriptome analysis of GRS and HG samples. Based on the findings at the transcriptional and metabolic levels, a mechanism model of the ginseng response to GRS was established. Our results provide new insights into ginseng’s response to GRS, which will reveal the potential molecular mechanisms of this disease in ginseng.

**Supplementary Information:**

The online version contains supplementary material available at 10.1186/s12870-021-03001-w.

## Background

Ginseng (*Panax ginseng* Mayer) is one of the most important foods and herbs because of its high nutritional value and medicinal potential. As a perennial plant, the active ingredients in ginseng accumulate over time [1]. Ginseng has higher requirements for the growing environment and a longer growing period, making it susceptible to various diseases during growth, thus affecting its yield and quality.

Ginseng rusty root symptoms (GRS) is one of the most common diseases in ginseng cultivation and production. It produces reddish-brown spots on the periderm of ginseng roots, and over time, the spots may gradually expand, leading to a decline in the commodity grade and quality of ginseng. Whether GRS is an infectious disease or a non-infectious physiological disease is still controversial, and some scholars in China have tried to answer this question [2, 3]. Previous research on American ginseng (*Panax quinquefolius L.*) showed that rusty roots might be a defence mechanism against the invasion of certain fungi, resulting in the stimulation of phenolic compound production [4, 5]. Some research results have indicated that fungal infection is the cause of GRS [6–8]. However, this disease’s effect on ginseng at the transcriptional and metabolic levels and the response mechanism of ginseng are still unclear.

RNA-seq is a method of transcriptome analysis using deep sequencing technology [9]. This method can detect almost all genes and pathways related to a physiological response with high sensitivity [10]. In recent years, increasing studies have used the Illumina RNA-eq platform based on transcriptome analysis to explore plant responses to abiotic or biological stresses and understand their associated molecular mechanisms [11–14]. Metabolomics, similar to transcriptomics, is an essential part of systems biology. Metabolomics aims to detect and quantify all metabolites in biological samples [15]. This method is gradually being applied in medicinal plant research and plant pathology as an essential bridge connecting genes, metabolites, and phenotypes. In the research field of plant pathology, this method has been gradually combined with other research methods and applied to research on plant disease diagnosis [16], plant disease resistance [17], plant noninfectious diseases [18, 19], and other fields showing good application prospects.

In this study, GRS and HG tissues from the same ginseng farm were selected as experimental materials. Transcriptome and metabolome analyses of GRS and HG tissues were performed using RNA-seq and ultra-high-performance liquid chromatography-tandem mass spectrometry (UHPLC–MS/MS) to elucidate the mechanism of the ginseng response to this disease. These findings provide not only valuable information for the prevention and treatment of GRS but also important insights for further understanding the molecular mechanism of the ginseng response to external stress.

## Results

### Quality control of RNA-seq reads

We performed RNA-seq analysis to compare the transcriptome between GRS and HG samples, and mainly analysed mRNA accumulation information obtained from the cDNA libraries. The sequencing of each ginseng sample produced over 100 million reads. After filtering out adaptors and low-quality reads, we obtained high-quality clean reads, accounting for more than 96% of the raw reads (Table S1).

### Differentially expressed genes (DEGs) of diseased tissues

**Fig. 1:**
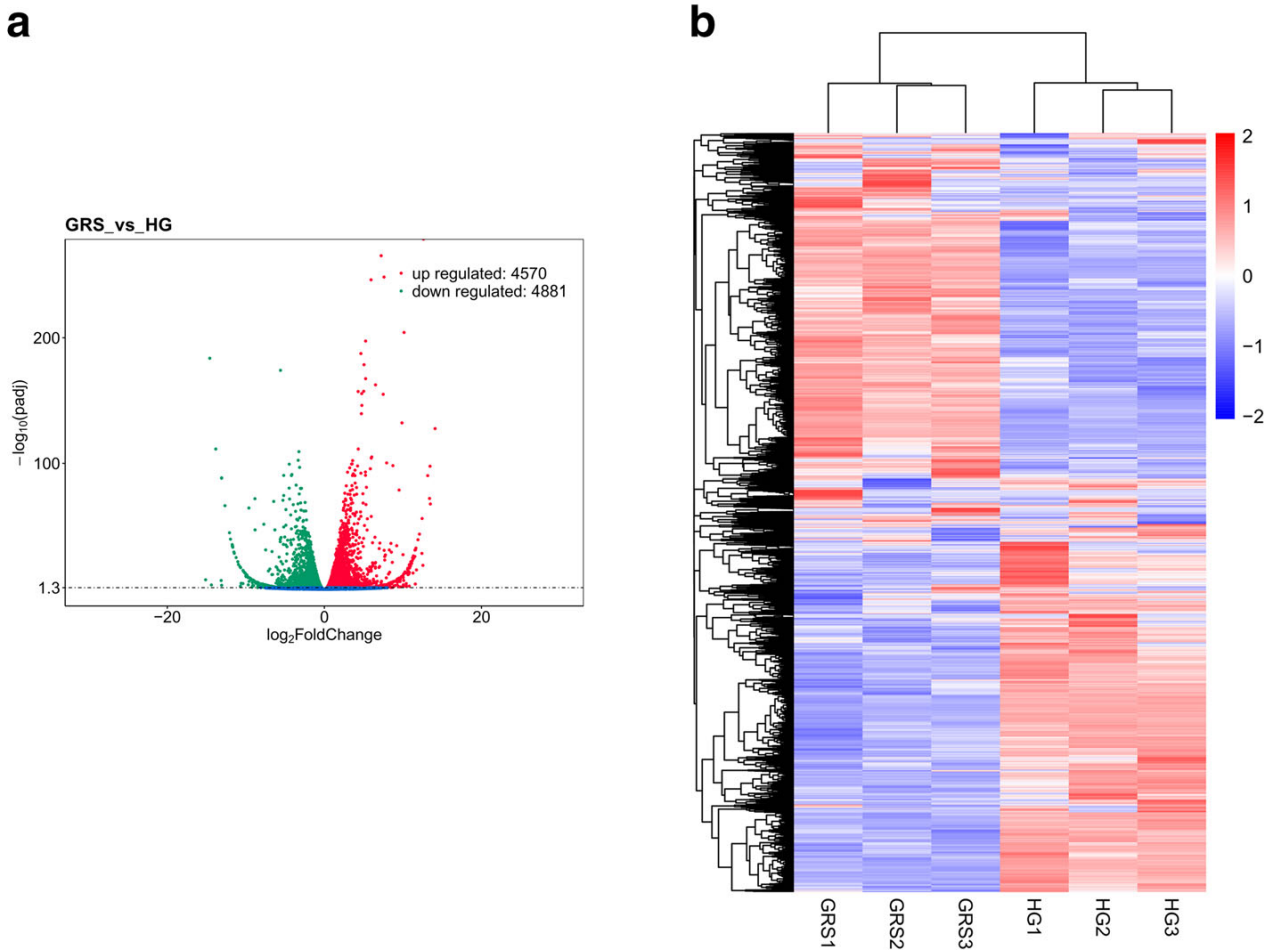
Expression profiling changes of genes in diseased tissues. Volcano plot indicating upregulated and downregulated genes (**a**); heat map showing hierarchical clustering of DEGs (**b**). HG: healthy ginseng; GRS: ginseng rusty root symptoms

The DEGs between the GRS and HG samples are shown in the volcano plot and clustering map (Fig. 1a-b). The results showed that compared with the HG group, the expression of 4570 genes in the GRS group was upregulated, and that of 4881 genes was downregulated.

To find the gene functions of DEGs in ginseng rusty roots, we performed Gene Ontology (GO) and Kyoto Encyclopedia of Genes and Genomes (KEGG) pathway analysis. GO is the international standard classification system for gene functions. All of the DEGs can be divided into three categories, including biological process (BP), cellular component (CC), and molecular function (MF).

**Fig. 2:**
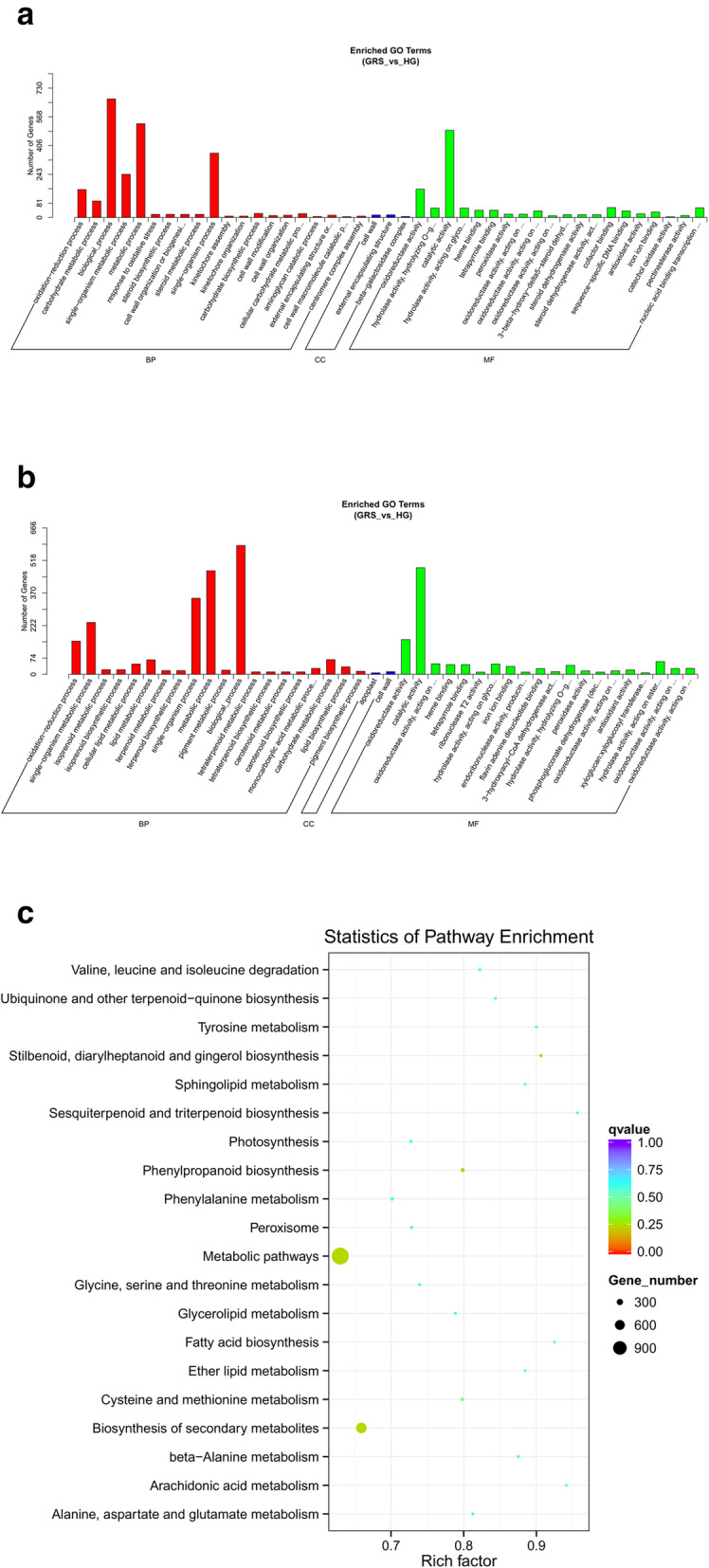
Functional GO and KEGG pathway classification of DEGs in diseased tissues. **a** GO analysis of upregulated genes; **b** GO analysis of downregulated genes; **c** DEGs enriched the KEGG pathway scatterplot

GO enrichment histograms drawn after classification of the enriched GO terms are shown in Fig. 2a. Based on GO analysis of the transcriptionally upregulated mRNAs, the most significantly enriched BPs were “carbohydrate metabolic process,” “steroid biosynthetic process,” “oxidation-reduction process,” “cell wall organization or biogenesis,” and “response to oxidative stress,” and the most significantly enriched CCs were “cell wall” and “beta-galactosidase complex.” The most significantly enriched MFs were “hydrolase activity,” “oxidoreductase activity,” and “peroxidase activity.” In addition, the histogram drawn according to transcriptionally downregulated mRNAs is shown in Fig. 2b. The results show that the most significantly enriched BPs were “oxidation-reduction process” and “terpenoid biosynthetic process,” and the most significantly enriched CCs were “apoplast” and “cell wall.” The most significantly enriched MFs were “iron ion binding,” “hydrolase activity,” and “peroxidase activity.” All of the enrichment results are shown in Tables S2 and S3.

For KEGG analysis of DEGs, the most significantly involved pathways were “metabolic pathways,” “biosynthesis of secondary metabolites,” and “phenylpropanoid biosynthesis” (Fig. 2c). All enriched KEGG pathways containing DEGs are given in Table S4.

### Differentially accumulated metabolites of diseased tissues

UHPLC–MS/MS determined the composition of metabolites in diseased and healthy tissues, and 1101 and 649 compounds were analysed in ginseng root tissues in the positive ion mode and negative ion mode, respectively (Tables S5-S6). The most abundant metabolites extracted included lipids and lipid-like molecules, organic acids and derivatives, alkaloids, and phenylpropanoids.

**Fig. 3:**
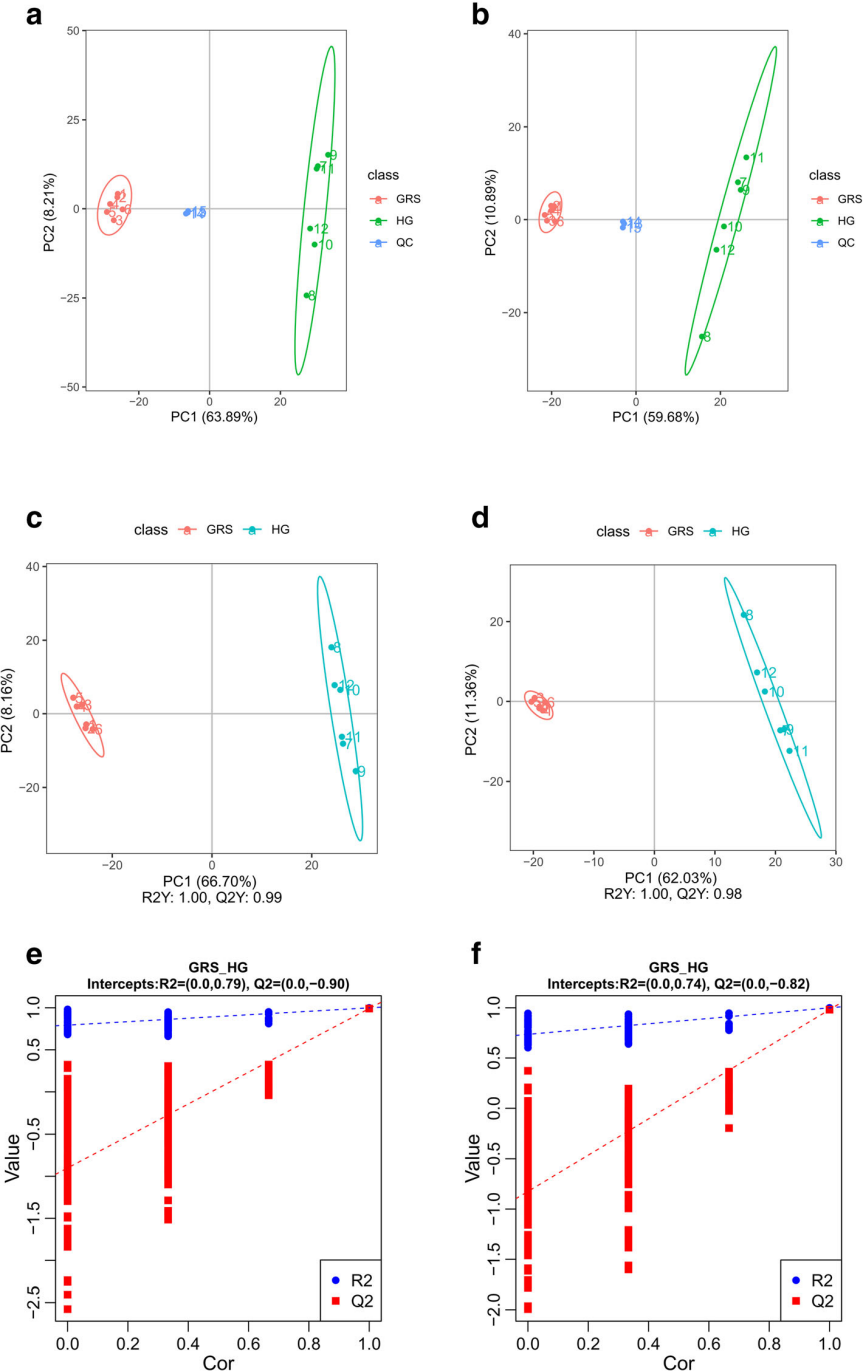
Metabolomics profiling of diseased and healthy ginseng tissues. PCA plots of the positive ion mode (**a**) and negative ion mode (**b**); PLS-DA plots of the positive ion mode (**c**) and negative ion mode (**d**); permutation tests for PLS-DA models in the positive ion mode (**e**) and negative ion mode (**f**). HG: healthy ginseng; GRS: ginseng rusty root symptoms

As shown in Fig. 3a-b for both the positive ion mode and negative ion mode, the metabolites for the two kinds of ginseng tissues were clustered together based on principal component analysis (PCA). This result suggests that there were significant differences in the metabolism of the two samples. We used the supervised partial least squares discriminant analysis (PLS-DA) method to separate the samples based on critical metabolite information. The PLS-DA score plot showed significant separation between the two groups of samples (Fig. 3c-d). Additionally, to evaluate the models’ reliability, we performed the permutation test with 200 iterations. The cross-validation results showed that neither the positive ion model nor the negative ion model was overfitted (R^2^ = 0.79 and Q^2^ = -0.90, R^2^ = 0.74 and Q^2^ = -0.82, respectively) (Fig. 3e-f).

**Fig. 4:**
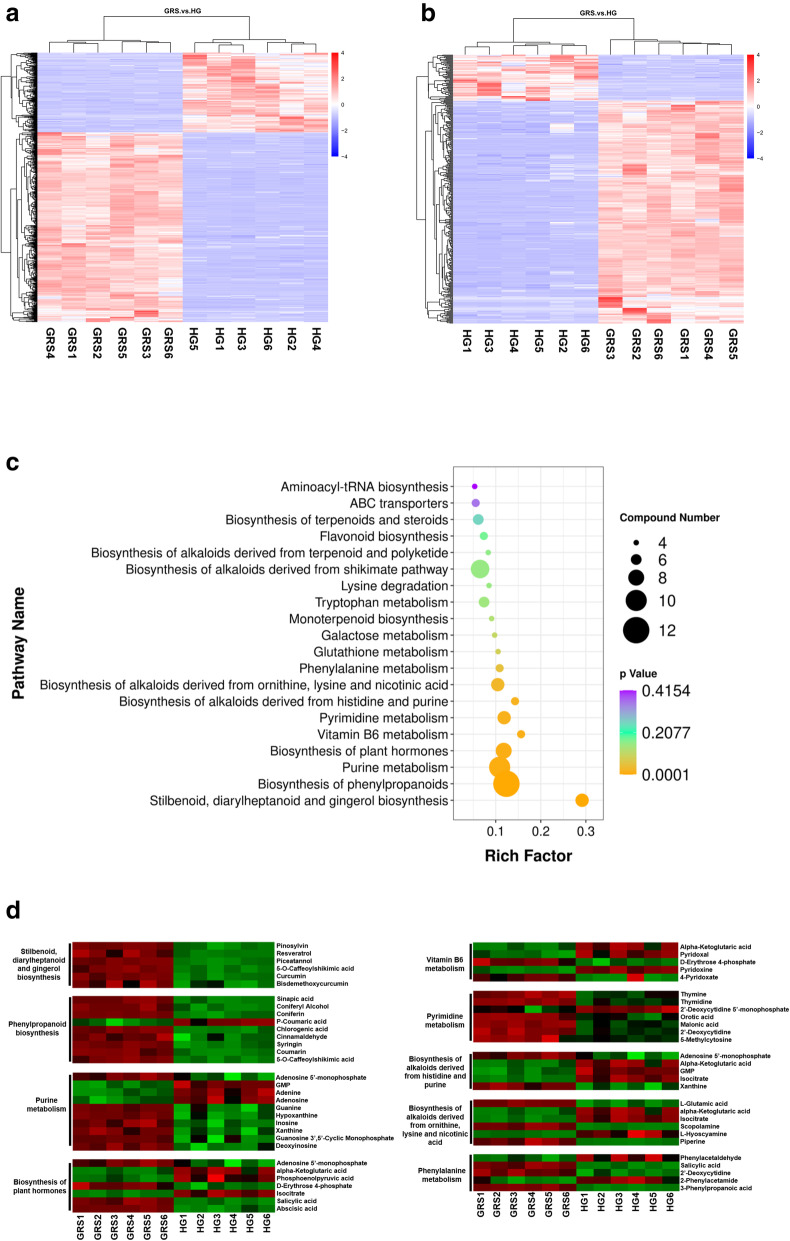
The hierarchical clustering analysis and heatmaps of differentially accumulated metabolites from diseased and healthy ginseng tissues identified in positive (**a**) and negative ion mode (**b**); differentially accumulated metabolites enriched the KEGG pathway scatterplot in the diseased tissues (**c**); changes in the accumulation of metabolites annotated to the KEGG metabolic pathway in ginseng rusty root symptoms tissues (**d**). HG: healthy ginseng; GRS: ginseng rusty root symptoms

We extracted VIP values from the variable importance plots of the PLS-DA models to search for differentially accumulated metabolites in diseased ginseng tissues. Differentially accumulated metabolites were screened based on three criterions: VIP > 1, *P* < 0.05 and fold change > 2. According to the above criteria, 595 and 344 metabolites were selected from the positive ion mode and negative ion mode, respectively. To better understand the chemical differences between diseased and healthy tissues, we plotted the heatmaps of 12 samples in positive and negative ion modes (Fig. 4a-b). Most of the differentially accumulated metabolites screened in positive ion mode had higher accumulation in diseased tissues (419 upregulated and 176 downregulated). The same phenomenon was observed in negative ion mode (285 upregulated and 59 downregulated). By matching these metabolites with those in the database, we finally identified 669 differentially accumulated metabolites, of which 508 were upregulated and 159 were downregulated (Table 1).

**Table 1:** Identification results of differential metabolites

	**Organic acids and derivatives**	**Alkaloids**	**Alcohols**	**Phenols**	**Phenylpropanoids**	**Terpene**	**Flavone**	**Lipids**	**Others**
up	95	54	60	53	47	39	43	29	30
down	36	32	10	4	7	13	7	15	6
	**Amino acids and derivatives**	**Strides**	**Carbohydrates**	**Quinones**	**Nucleotide and derivatives**	**Vitamins and derivatives**	**Indole derivatives**	**Plant hormones**	**Amides**
up	25	9	4	6	5	2	4	3	2
down	8	8	5	1	2	3	0	0	0

The differentially accumulated metabolites in the two tissues were mapped to the KEGG database. These metabolites were mainly enriched in multiple alkaloid biosynthesis pathways, such as “Biosynthesis of alkaloids derived from histidine and purine” and “Biosynthesis of alkaloids derived from ornithine, lysine and nicotinic acid.” In addition, the extremely significant differences in “Stilbenoid, diarylheptanoid and gingerol biosynthesis,” “Phenylpropanoid biosynthesis,” “Biosynthesis of plant hormones,” and “Purine metabolism” are also noteworthy (Fig. 4c). The abundances of differentially accumulated metabolites across 9 metabolic pathways, as well as the significantly enriched differentially accumulated metabolites in KEGG metabolic pathways and those with a compound number ≥ 5 are shown in Fig. 4d.

### Response of ginseng to disease

The results of the GO and KEGG enrichment analyses showed the response of GRS tissues. Numerous DEGs in GRS tissues were involved in plant hormone signal transduction, lignin synthesis, plant-pathogen interactions, and oxidative stress compared with those in HG tissues.

We focused on the pathway “plant hormone signal transduction,” involving 135 DEGs (Fig. S1-S2). Almost all genes involved in ABA signal transduction, such as soluble ABA receptors PYL/RCAR, protein phosphatases type-2C (PP2Cs), and SNF1-related protein kinase (SnRK2.3, SnRK2.10), were upregulated in the GRS group (Fig. S1B). The JAR1 and coronatine insensitive 1 (COI1) genes involved in JA signalling were upregulated, and the jasmonate-zim domain (JAZ) gene was downregulated (Fig. S1C). Additionally, differential gene expression of mediator protein nonexpressor of pathogen-related gene 1 (NPR1), TGA factors, and pathogenesis-related gene PR-1 involved in SA signalling was found for GRS (Fig. S1D). The DEGs associated with plant hormone signal transduction are shown in Fig. S2. In addition, we found that methyl jasmonate (MeJA), abscisic acid (ABA), and salicylic acid (SA) were strongly induced in diseased tissues (Fig. S1A).

**Fig. 5:**
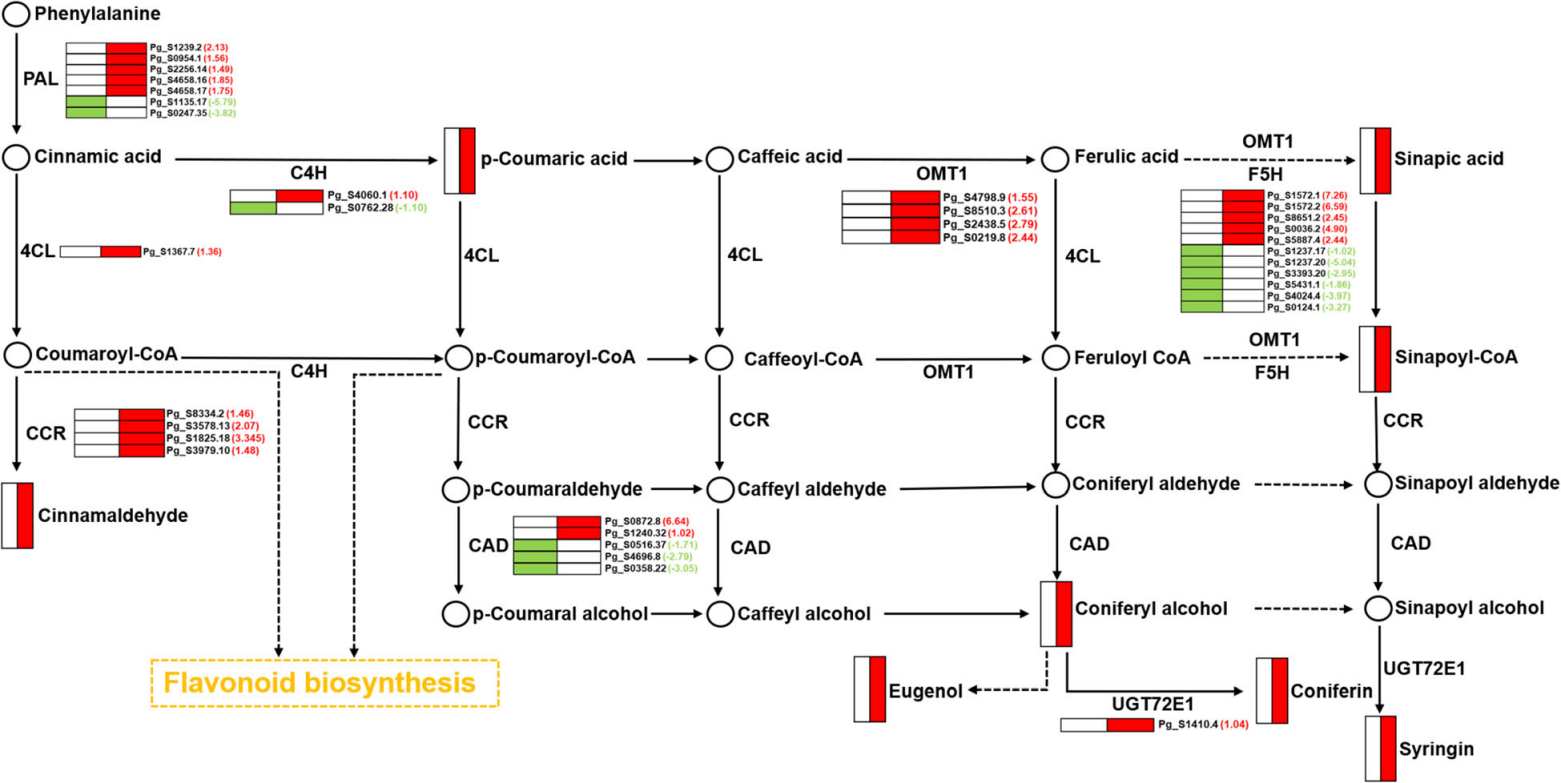
DEGs and differentially accumulated metabolites related to phenylpropanoid biosynthesis. The different color indicates the DEGs and differentially metabolites in GRS compared with HG (Red frame: up-regulation; green frame: down-regulation). PAL, phenylalanine ammonia-lyase; C4H, cinnamate 4-hydroxylase; 4CL, 4-coumarate: CoA ligase; CCR, cinnamoyl-CoA reductase; CAD, cinnamyl alcohol dehydrogenase; OMT1, caffeic acid 3-Omethyltransferase 1; F5H, ferulate-5-hydroxylase

There were 124 DEGs and 9 differentially accumulated metabolites involved in the “Phenylpropanoid biosynthesis” pathway (Table S4 and Fig. 4d). The detailed locations of differentially accumulated metabolites and DEGs in the pathway are shown in Fig. 5. In GRS tissues, enzymes closely related to lignin and phenol synthesis, such as phenylalanine ammonia-lyase (PAL), O-methyltransferase 1 (OMT1), 4-coumarate: coenzyme A ligase 1 (4CL1), and cinnamoyl coenzyme A reductase (CCR), were detected with a high expression level.

We analysed the “peroxisome” pathway, which is closely related to oxidative stress in plants. As shown in Fig. S3 and Fig. S4, we found that 59 DEGs were involved in the “peroxisome” pathway. Several PEX family genes, such as PEX14, PEX16, and PEX19, were downregulated in the GRS group. PXMP2 and MPV17 were involved in reactive oxygen species (ROS) metabolism and showed obvious downregulated expression. Additionally, catalase (CAT) and superoxide dismutase (SOD) in the antioxidant system were upregulated.

In our enrichment results, 96 DEGs were involved in the “plant-pathogen interaction” pathway (Table S4). The corresponding KEGG pathway is shown in Fig. S5. Several genes, including the respiratory burst oxidase homologue (RBOH) genes RBOHD and RBOHF, flagellin sensing 2 (FLS2), and WRKY family genes (WRKY33 and WRKY22), involved in pathogen-associated molecular pattern (PAMP)-triggered immunity (PTI) were differentially expressed. The RIN4, RPS2, and HSP genes related to effector-triggered immunity (ETI) were also differentially expressed. Furthermore, the defence-related genes NHO1 and PR1 were upregulated (Fig. S6).

### Correlation analysis of differentially accumulated metabolites and DEGs

**Fig. 6:**
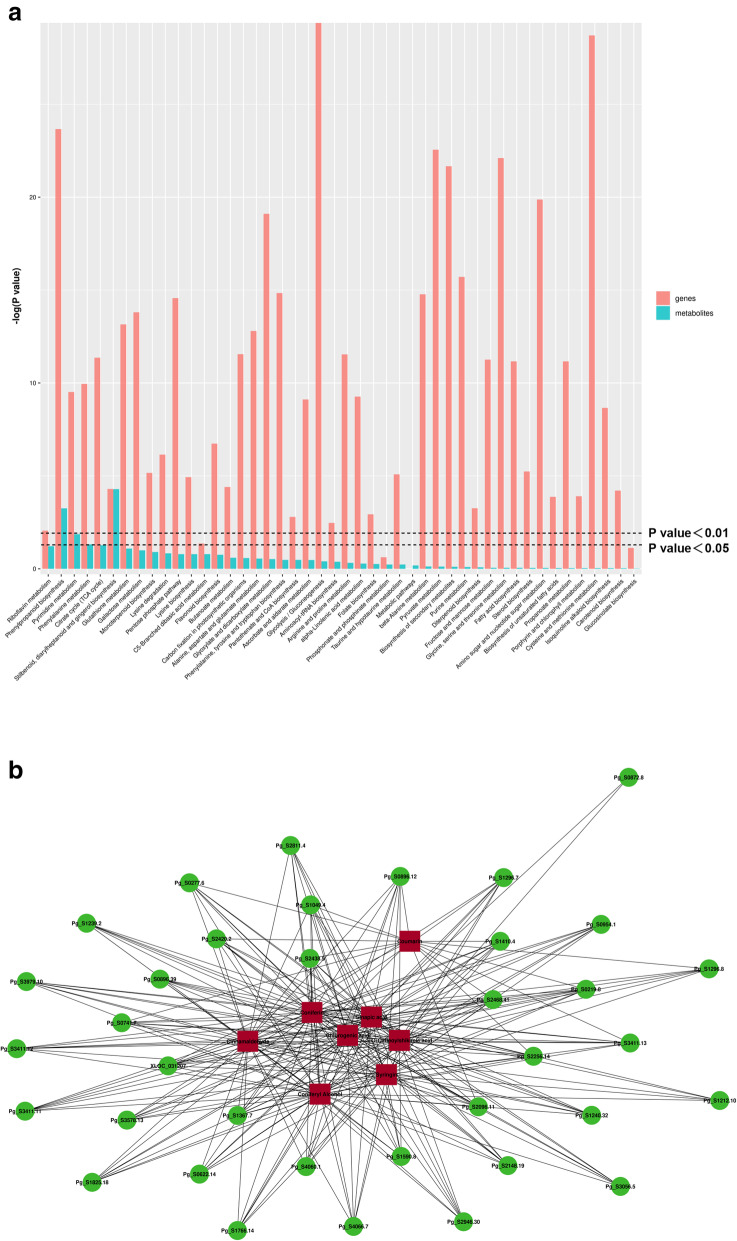
**a** Statistical results of DEGs and metabolites enriched in the same KEGG pathway; **b** connection network between DEGs and differentially accumulated metabolites in phenylpropanoid biosynthesis pathway

We found that many DEGs and differentially accumulated metabolites were enriched in the same KEGG metabolic pathways in both types of tissue. These pathways include “Phenylpropanoid biosynthesis,” “Phenylalanine metabolism,” “Stilbenoid, diarylheptanoid and gingerol biosynthesis,” and others (Fig. 6a). In addition, considering the role of the phenanthrene biosynthesis pathway in disease resistance and the stress response in plants, we constructed a correlation network graph of DEGs and differentially accumulated metabolites based on the Pearson correlation coefficient (PCC). As shown in Fig. 6b, a total of 36 DEGs and 8 differentially accumulated metabolites in the network were highly positively correlated (PCC > 0.8). It is speculated that these genes and metabolites may play a key role in the response of ginseng to stress.

### Real-time quantitative polymerase chain reaction (qPCR) validation of mRNA expression

**Fig. 7:**
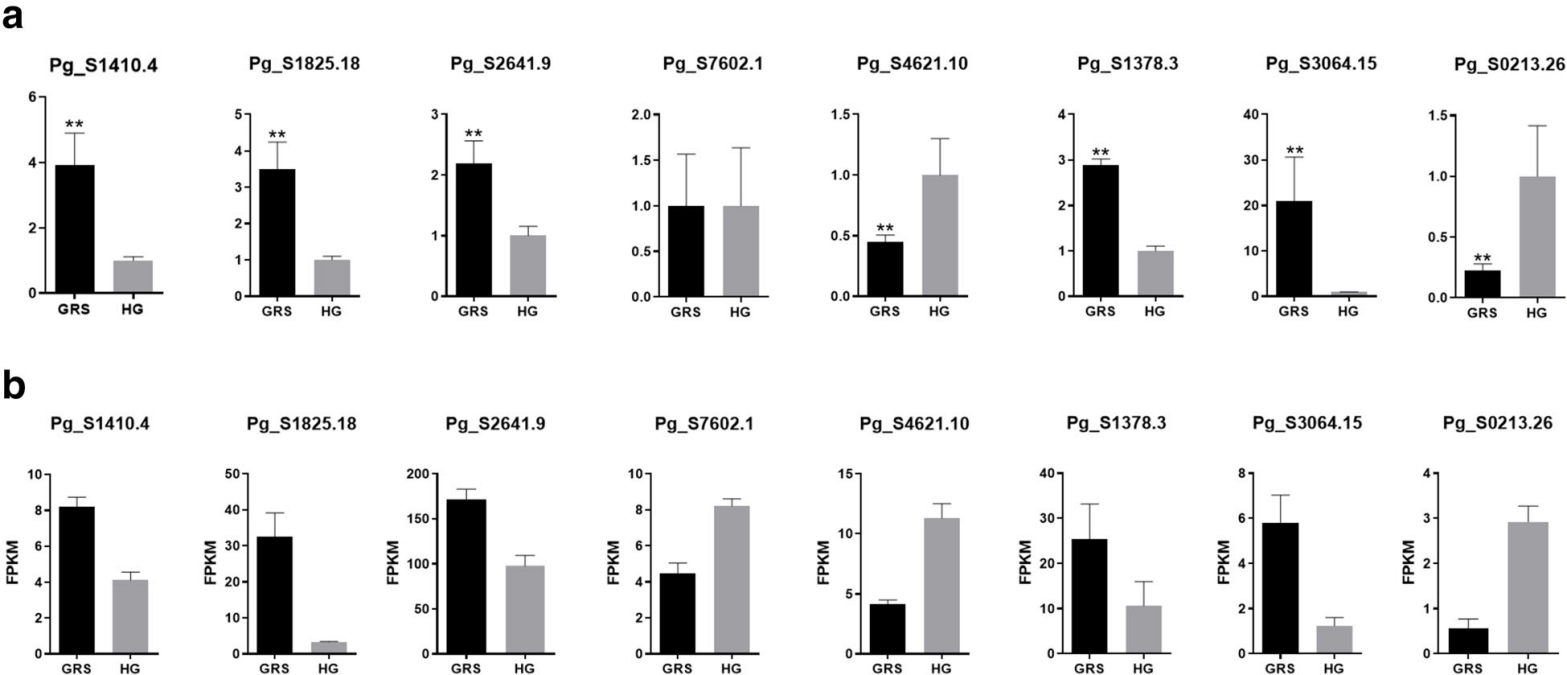
qRT-PCR validation of significant DEGs. **a** qRT-PCR, the data were presented as the mean ± SEM (*n* = 3); **p < 0.05; **b** RNA-seq. HG: healthyginseng; GRS: ginseng rusty root symptoms

To verify the accuracy of the RNA-seq results and provide the basis for further research, some genes from the four plant resistance pathways were selected for qRT-PCR analysis. The expression level of these genes was demonstrated by qRT-PCR and transcriptome sequencing. The genes Pg_S1410.4 (UGT72E1), Pg_S1825.18 (CCR1), Pg_S2641.9 (CSD1), Pg_S7602.1 (MPV17), Pg_S4621.10 (FLS2), Pg_S1378.3 (HSP90.1), Pg_S3064.15 (RP-1), and Pg_S0213.26 (JAZ) were analysed by qPCR (Fig. 7a). The expression levels of the corresponding mRNAs obtained by RNA-seq are shown in Fig. 7b. All validation results fully proved the reliability and accuracy of the transcriptome sequencing data.

## Discussion

**Fig. 8:**
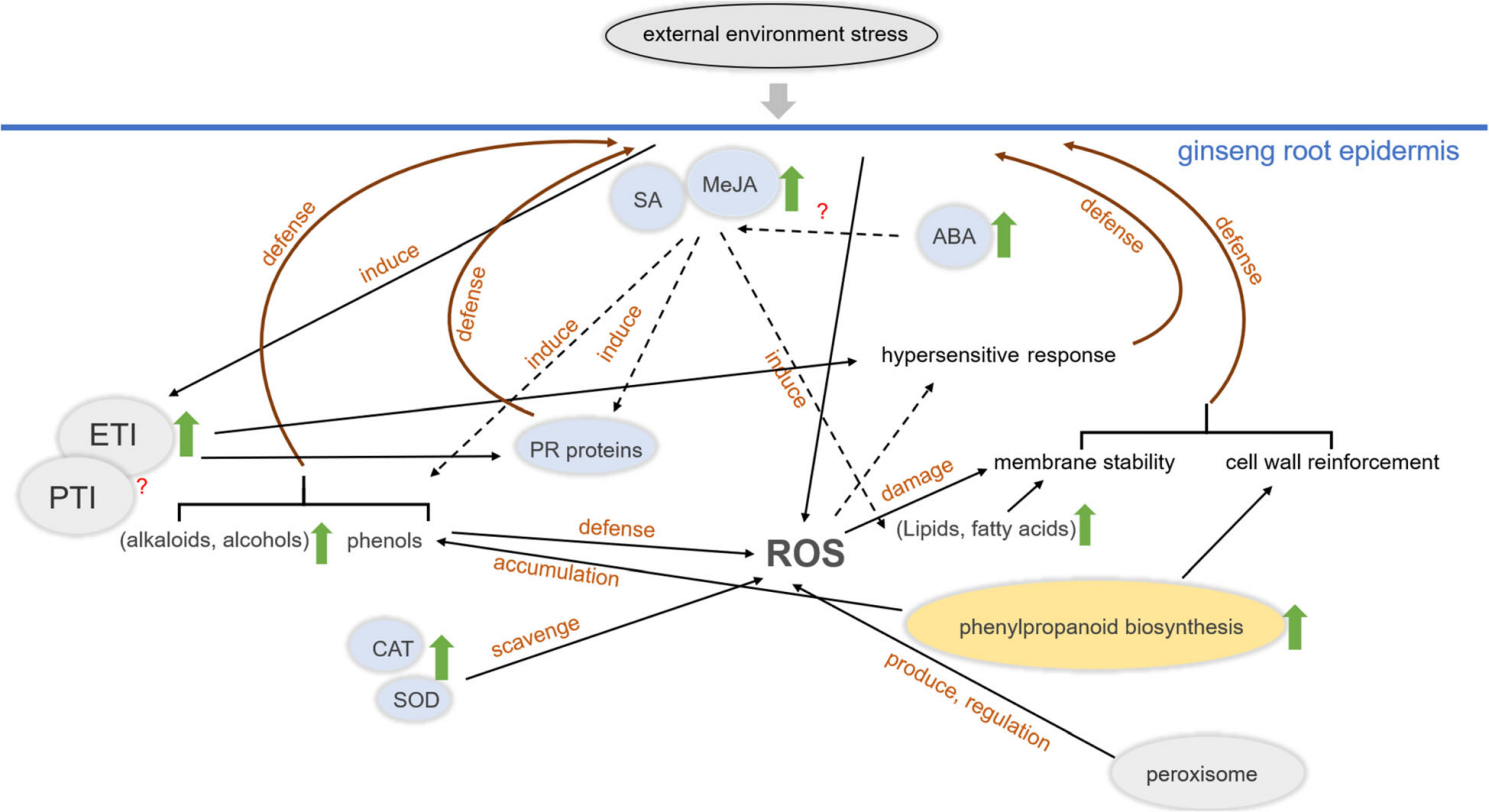
Response mechanism of ginseng to rusty root symptoms. “?” means not validated; The solid arrows indicate the direct action and the dotted arrows indicate the indirect action. Environmental stress induces oxidative stress, which activates the ETI system and stimulates the synthesis of SA, MeJA, and ABA in ginseng tissues. SA and MeJA induce the accumulation of phenolic, alkaloid, and alcohol metabolites. SA and MeJA also induce lipid and fatty acid metabolism and enhance membrane stability against stress. The production of antioxidant enzymes such as CAT and SOD and the peroxisome regulation affect ROS accumulation in tissues. Meanwhile, DEGs in PTI and ETI in ginseng epidermal tissues may promote PR proteins' production and hypersensitive responses against external stresses. The activation of the phenylpropanoid biosynthesis pathway enhances the production of phenolic metabolites on the one hand. It may increase the accumulation of lignin and promote the cell wall's reinforcement on the other hand. The reinforcement of the cell wall and the enhancement of membrane stability together constitute a physical defense in ginseng tissues

Metabolites and total RNAs were extracted from the same variety of 5-year-old ginseng planted in the same ginseng farm (due to its higher rusty root index). The samples were prepared from healthy and diseased ginseng tissues. The purpose was to ensure that ginseng samples experienced the same field management and climate changes during the growth process [20]. We conducted UHPLC–MS/MS and RNA-seq analysis of GRS and HG tissues. The results showed that in the GRS sample, 939 metabolites were differentially accumulated, and 9451 genes were significantly differentially expressed. Based on previous studies and the results of this metabolomic and transcriptomic analysis, we propose a model to explain ginseng’s response to GRS (Fig. 8).

### Hormone regulation in diseased tissues

Plants produce a variety of hormones during growth. ABA, SA, and JA play an essential role in mediating plant defence responses to pathogens and abiotic stresses [21, 22]. At the transcriptional level, the ABA receptor PYR/PYL/RCAR, negative regulator PP2C, and positive regulator SnRK2 together comprise the regulatory system [23]. In GRS tissues, the genes that regulate ABA signal transduction were differentially expressed, and ABF transcription factors were upregulated (Fig. S1B). Under JA-stimulated conditions, JA-Ile, synthesized by JAR1, binds to the receptor COI1, leading to the degradation of the repressor protein JAZ, which upregulates the expression level of JA target genes (Fig. S1C) [24, 25]. NPR1 is one of the key regulatory elements of SA-dependent PR gene activation and interacts with GTA factors to regulate PR gene expression [26, 27]. We found differential expression of genes that are by-products of NPR1 (BOP2 and NPR3) and multiple TGA transcription factors genes, which resulted in the upregulation of PR-1 gene expression (Fig. S1D). In addition, three plant hormones and hormone-related signalling molecules (ABA, SA, and MeJA) were detected by UHPLC-MS/MS, and all of them were significantly upregulated in diseased tissues (Fig. S1A). Therefore, the accumulation of hormones and the differential expression of genes related to hormone signal transduction in diseased tissues affected metabolic processes and eventually led to changes in growth patterns to withstand environmental stress.

### Immune system in diseased tissues

Plants have two layers of defence against pathogens, PTI and ETI [28]. Here, compared to HG tissues, there were 96 DEGs associated with the plant-pathogen interaction pathway in GRS tissues. RBOH mediates ROS generation, which is strongly related to both PTI and ETI [29, 30]. RBOHD and RBOHF are considered to be critical components of plant defences [31]. FLS2 is a pattern recognition receptor (PRR) that can trigger innate immunity in plants and is also involved in endocytosis [32]. WRKY33 plays a vital role as a transcription factor in plant resistance to pathogens [33]. The downregulation of the above genes, along with NOA1 and MEKK1, may indicate that part of PTI is repressed during GRS.

Interestingly, ETI seems to be activated in GRS samples. RPS2 is a resistance (R) protein in plants, while RIN4 is an essential negative regulator of natural plant immunity and has a regulatory effect on RPS2 [34]. HSP90.1 is required for RPS2 resistance and is rapidly induced in plants in the face of biological stress [35]. SGT1 positively regulates disease resistance produced by many R proteins [36]. In our analysis, RIN4 was downregulated, and PRS2, SGT1, and HSP90.1 were upregulated. Furthermore, NHO1, an essential universal resistance gene in plants, was upregulated [37]. Overall, the immune system of diseased tissues was activated, which indirectly led to hypersensitivity and cell wall reinforcement.

### ROS-scavenging system in diseased tissues

Exposure of plants to various biotic and abiotic stress conditions triggers rapid changes in reactive oxygen species (ROS) production and clearance [38]. ROS play an essential role in signalling pathways that regulate inflammatory and defence responses in plants, but their accumulation is often harmful to cells.

Peroxisomes are subcellular organelles with a basic oxidative type of metabolism and may be the primary intracellular ROS production site [39, 40]. KEGG enrichment analysis showed that both PXMP2 and MPV17, which are involved in ROS metabolism, were transcriptionally downregulated (Fig. S2). PXMP2 is a perovskite membrane protein with a channel-forming activity that allows free diffusion of compounds, such as H_2_O_2_ [41]. MPV17 is a protein involved in the production of ROS [40]. Several PEX family protein genes were differentially expressed, including PEX13 and PEX14, which are involved in matrix protein import, and PEX16 and PEX19, which are involved in membrane protein import [42, 43]. In addition, CAT and SOD genes were upregulated in diseased tissues. Thus, diseased tissues may respond to oxidative stress through peroxisomes and antioxidant systems [44].

The phenolic content is often considered to be closely related to plant tissues’ total antioxidant capacity [45]. Studies have shown that flavonoids are also one of the main chemical defence substances in plants and can reduce various forms of reactive oxygen species in plant cells [46]. In our results, a significant upregulation of a large number of flavonoids and phenolics was found. Significant enrichment of the flavonoid biosynthetic pathway was also found by KEGG enrichment analysis (Fig. 4c). We also noted that multiple alcohol metabolites showed higher accumulation in diseased tissues. It was shown that the accumulation of alcohol usually plays a role in the fight against oxidative stress. Alcohols can act as intermediates of redox reactions with good scavenging capacity for free radicals and superoxide [47, 48]. It can be speculated that ginseng accumulates more alcohols in diseased tissues to cope with oxidative stress to improve its resistance. Overall, these compounds accumulate in diseased tissues of ginseng and may be jointly involved in the oxidative stress response.

### Physical and chemical defence in diseased tissues

Lignin is a highly branched polymer of phenylpropanoid compounds and is the main component of the plant cell wall [49]. Lignin biosynthesis involves a complex genetic network containing many containing enzymes [50, 51]. Several key enzymes, including PAL, 4CL, CCR, OMT, and CAD, were upregulated [52, 53]. In addition, phenols and flavonoids not only play a key role in the plant antioxidant process, but are also essential compounds involved in plant chemical defences [54]. Our analysis showed that both types of data (transcriptomic and metabolomic) were significantly enriched in the phenylpropanoid biosynthesis pathway (Fig. 6a). Considering that the phenylpropanoid biosynthesis pathway is closely related to phenolic production, flavonoid metabolism, and lignin formation, it is speculated that it may be a critical metabolic pathway in ginseng in response to GRS [55]. Therefore, we constructed a correlation network of differentially accumulated metabolites and DEGs in this metabolic pathway based on the correlation analysis results (Fig. 6b). The network includes several upstream regulatory genes of phenolic, lignin, and flavonoid synthesis, such as PAL (Pg_S1239.2, Pg_S0954.1, and Pg_S2256.14), 4CL (Pg_S1367.7), and C4H (Pg_S4060.1), and precursor compounds, such as coniferin, sinapic acid, and coumarin. It is speculated that the upregulation of these genes and the accumulation of metabolites play a crucial role in the ginseng response to GRS.

Lipids are essential membrane components, and changes in their composition may help plants maintain cellular compartmentalization and membrane integrity [56]. In addition, free radicals are metabolized and produced when cells are under stress or in the process of injury. Therefore, reducing lipid peroxidation has an essential role in plant resilience. In diseased tissues of ginseng, there were more lipid metabolites with significantly altered contents. For example, phosphatidylcholine (PE) and phosphatidylethanolamine (PC) are lipids that are present in large amounts in cell membranes. An increase in the PC/PE ratio has been reported to be a marker of the membrane bilayer’s structural stability to prevent membrane degradation [57]. Interestingly, we detected a significant increase in several PC levels and a substantial decrease in PE levels in diseased tissues of ginseng. Therefore, diseased tissues may have a stronger tendency to maintain biofilm integrity and stability than healthy tissues.

The accumulation of alkaloids is a vital defence strategy for plants in response to biotic stresses, and alkaloids are a crucial class of compounds involved in plant chemical defences [58]. In diseased tissues, we found an upregulation of the accumulation of numerous alkaloid metabolites. The KEGG enrichment results of differentially accumulated metabolites also contained numerous alkaloid biosynthetic pathways (Fig. 2c). This result suggests that alkaloid metabolites are essential for the stress response of ginseng. Chemical defences against external stress involving alkaloids may occur in diseased tissues [55].

## Conclusions

The current study performed a comparative metabolome and transcriptome analysis of GRS and HG tissues. An analysis of the pathways associated with the plant resistance response revealed that hormone signalling pathways, lignin biosynthesis, ROS regulation by peroxisomes changes during the plant-pathogen response. Additionally, the diseased tissues’ metabolic patterns changed, and the production and accumulation of many secondary metabolites may play an essential role in GRS. Finally, a model was proposed to explain the response of ginseng to GRS (Fig. 8). Our results provide new insights into ginseng’s response to GRS, revealing the potential molecular mechanisms of this disease in ginseng.

## Methods

### Plant material and tissue collection

All ginseng samples (5 years old) were collected from the same ginseng farm in Hunchun city, Jilin Province, China (42.86′N and 130.37′E). In the previous survey, this was a ginseng farm with a high rusty root index [59].

GRS diseased tissues (main root) and HG healthy tissues were collected as samples frozen in liquid nitrogen immediately and stored at − 80 °C before use. Independent biological replicates were prepared, and each replicate included root materials from three or more ginseng plants.

### RNA extraction, library preparation, clustering and sequencing

Six complementary DNA (cDNA) libraries, three for GRS tissues, and three for HG tissues, were constructed. TRIzol reagent (Invitrogen, Carlsbad, CA) was used to isolate each sample’s total RNA. RNA degradation and contamination were monitored on 1% agarose gels. RNA purity was checked using a NanoPhotometer® spectrophotometer (IMPLEN, CA, USA). RNA concentration was measured using Qubit® RNA Assay Kit in a Qubit® 2.0 Fluorometer (Life Technologies, CA, USA). RNA integrity was assessed using an RNA Nano 6000 Assay Kit and the Bioanalyzer 2100 system (Agilent Technologies, CA, USA).

A total amount of 3 μg of RNA per sample was used as the input material for RNA sample preparation. Sequencing libraries were generated using an NEBNext® Ultra™ RNA Library Prep Kit for Illumina® (NEB, USA), following the manufacturer’s recommendations, and index codes were added to attribute sequences to each sample. Briefly, mRNA was purified from the total RNA using poly-T oligo-attached magnetic beads. Fragmentation was conducted using divalent cations under elevated temperatures in NEBNext First Strand Synthesis Reaction Buffer (5X). First strand cDNA was synthesized using a random hexamer primer and M-MuLV Reverse Transcriptase (RNase H-). Second strand cDNA synthesis was subsequently performed using DNA Polymerase I and RNase H. The remaining overhangs were converted into blunt ends via exonuclease/polymerase activities. After adenylation of the 3′ ends of DNA fragments, the NEBNext adaptor with a hairpin loop structure was ligated to prepare for hybridization. To preferentially select cDNA fragments that were 150–200 bp in length, the library fragments were purified with the AMPure XP system (Beckman Coulter, Beverly, USA). Then, 3 μl of USER Enzyme (NEB, USA) was used with size-selected, adaptor-ligated cDNA at 37 °C for 15 min followed by 5 min at 95 °C before PCR. PCR was performed with Phusion High-Fidelity DNA polymerase, universal PCR primers, and an index (X) primer. PCR products were purified (AMPure XP system), and library quality was assessed on the Agilent Bioanalyzer 2100 system.

After cluster generation, RNA library preparations were sequenced on an Illumina HiSeq PE150 platform, and 150 bp paired-end reads were generated. The raw RNA-seq data are freely available in the NCBI database under accession no. PRJNA684799.

### Transcriptome data analysis

Raw data (raw reads) in fastq format were first processed through in-house Perl scripts. A certain length range was then chosen from clean reads to perform all of the downstream analyses.

The reference genome and gene model annotation files were downloaded from the genome website directly [60]. The reference genome index was built using STAR, and paired-end clean reads were aligned to the reference genome using STAR (v.2.5.1b). STAR used the maximal mappable prefix (MMP) method to generate a precise mapping result for junction reads. HTSeq (v.0.6.0) was used to count the number of reads mapped to each gene. The expected number of fragments per kilobase of transcripts per million mapped reads (FPKM) of each gene was calculated based on the length and mapped read count for that gene.

Differential expression analysis of the two groups was performed using the DESeq2 R package (v.1.10.1). The resulting *P* values were adjusted using Benjamini and Hochberg’s approach for controlling the false discovery rate.

GO (http://www.geneontology.org/) enrichment analysis of DEGs was implemented by the GOseq-based Wallenius noncentral hypergeometric distribution, in which gene length bias was corrected [61]. In addition, we performed KEGG enrichment analysis on DEGs using KOBAS 2.0 software [62].

### Metabolome analysis

First, we extracted the metabolites. Tissues (100 mg) were individually ground with liquid nitrogen, and the homogenate was resuspended in prechilled 80% methanol and 0.1% formic acid by vortexing. The samples were incubated on ice for 5 min and then were centrifuged at 15,000 rpm and 4 °C for 5 min. Some supernatants were diluted with UHPLC–MS/MS grade water to a final concentration of 53% methanol. The samples were subsequently transferred to a fresh Eppendorf tube and then centrifuged at 15000×g and 4 °C for 10 min.

UHPLC-MS/MS analysis was then performed using a Vanquish UHPLC system (Thermo Fisher, Germany) coupled with an Orbitrap Q Exactive™ HF-X mass spectrometer (Thermo Fisher, Germany). Samples were injected onto a Hypesil Gold column (100 × 2.1 mm, 1.9 μm) using a 17-min linear gradient at a flow rate of 0.2 mL/min. The eluents for the positive polarity mode were eluent A (0.1% FA in water) and eluent B (methanol). The eluents for the negative polarity mode were eluent A (5 mM ammonium acetate, pH 9.0) and eluent B (methanol). The solvent gradient was set as follows: 2% B, 1.5 min; 2–100% B, 12.0 min; 100% B, 14.0 min; 100–2% B, 14.1 min; 2% B, 17 min. A Q Exactive™ HF-X mass spectrometer was operated in positive/negative polarity mode with a spray voltage of 3.2 kV, capillary temperature of 320 °C, sheath gas flow rate of 40 arb, and aux gas flow rate of 10 arb.

The raw data files generated by UHPLC-MS/MS were processed using the Compound Discoverer 3.1 (CD3.1, Thermo Fisher) to perform peak alignment, peak selection, and quantitation for each metabolite. The main parameters were set as follows: retention time tolerance, 0.2 min; actual mass tolerance, 5 ppm; signal intensity tolerance, 30%; signal/noise ratio, 3; and minimum intensity, 100,000. After that, peak intensities were normalized to the total spectral intensity. The normalized data were used to predict the molecular formula based on additive ions, molecular ion peaks, and fragment ions. Then, peaks were matched with the mzCloud, mzVault, and MassList databases to obtain accurate qualitative and relative quantitative results.

PCA and PLS-DA were performed using SIMCA-P 14.1 (Umetrics, Umea, Sweden). The metabolites with a VIP > 1, *P*-value < 0.05 and fold change > 2 were considered to be differential metabolites. KEGG enrichment analysis of differentially accumulated metabolites was performed using KOBAS 2.0 software [62].

### Combined analysis of DEGs and differentially accumulated metabolites

The obtained differentially accumulated metabolites and DEGs were mapped to KEGG pathway for integration. In addition, we performed correlation analysis on transcriptomic and metabolomic data to explore correlations between DEGs and metabolites. The PCC of DEGs and differentially accumulated metabolites was calculated using the COR function in R. The genes and metabolites with a PCC > 0.8 in the KEGG pathway were used to establish the related network that was visualized by Cytoscape software.

### Quantitative real-time qPCR

Quantitative PCR was performed on a Real-Time PCR System (Lightcycler 96, Roche, Switzerland) using 2 × RealStar Green Fast Mixture (GenStar, China). For each reaction, 0.5 μl of the forward and reverse primers and 2 μl of the cDNA template were added. All of the primers used in this study are listed in Table S7. The relative gene expression level was calculated according to the 2^-ΔΔCt^ method [63].

### Statistical analysis

Statistical analysis was performed with SPSS 20.0 software. All of the data were expressed as the mean ± SEM. *P* < 0.05 was considered statistically significant. RT-PCR results were analysed using a Student’s *t*-test. Significance was established at *P* < 0.05.

## Supplementary Information

**Additional file 1: Table S1.** Summary of RNA-seq data and mapped reads for mRNA.

**Additional file 2: Table S2.** Gene Ontology results of all the enriched terms for the upregulated mRNAs in GRS tissues.

**Additional file 3: Table S3.** Gene Ontology results of all the enriched terms for the downregulated mRNAs in GRS tissues.

**Additional file 4: Table S4.** All pathways associated with DEGs based on KEGG enrichment analysis.

**Additional file 5: Table S5.** Results of UHPLC–MS/MS analysis in positive ion mode.

**Additional file 6: Table S6.** Results of UHPLC–MS/MS analysis in negative ion mode.

**Additional file 7: Table S7.** Primer sequences of the genes for qPCR verification.

**Additional file 8: Figure S1.** Plant hormone contents and heatmaps of DEGs.

**Additional file 9: Figure S2.** The plant hormone signal transduction pathway.

**Additional file 10: Figure S3.** Heatmap of DEGs related to peroxisome synthesis.

**Additional file 11: Figure S4.** The peroxisome pathway.

**Additional file 12: Figure S5.** The plant-pathogen interaction pathway.

**Additional file 13: Figure S6.** Heatmap of DEGs related to plant-pathogen interaction.

## Data Availability

All relevant supporting data sets are included in the article and its supplemental files.

## Abbreviations

GRS: Ginseng rusty root symptoms
HG: Healthy ginseng
UHPLC–MS/MS: Liquid chromatography-tandem mass spectrometry
KEGG: Kyoto Encyclopedia of Genes and Genomes
DEGs: Differentially expressed genes
GO: Gene Ontology
BP: Biological process
CC: Cellular component
MF: Molecular function
PCA: Principal component analysis
OPLS-DA: Partial least-squares discriminant analysis
MeJA: Methyl jasmonate
ABA: Abscisic acid
SA: Salicylic acid
PP2Cs: Protein phosphatases type-2C
SnRK: SNF1-Related Protein Kinase
COI1: Coronatine insensitive 1
JAZ: Jasmonate-zim domain
NPR1: Protein non-expressor pr gene 1
PAL: Phenylalanine ammonia-lyase
OMT1: O-methyltransferase1
4CL: 4-coumarate: coenzyme A ligase
CCR: Cinnamoyl coenzyme A reductase
ROS: Reactive oxygen species
CAT: Catalase
SOD: Superoxide dismutase
RBOHs: Respiratory burst oxidase homologues
FLS2: Flagellin sensing 2
PAMP: Pathogen-associated molecular patterns
PTI: PAMP-triggered immunity
ETI: Effector-triggered immunity
PPC: Pearson correlation coefficient
qPCR: Quantitative polymerase chain reaction
cDNA: Complementary DNA
MMP: Maximal mappable prefix
FPKM: Fragments per kilobase of transcript per million mapped reads
