# Supplementary material for: Metabolome and transcriptome analysis reveals the molecular profiles underlying the ginseng response to rusty root symptoms
Source: BMC Plant Biol. 2021 May 13;21:215. doi: 10.1186/s12870-021-03001-w (PMC8117609; doi:10.1186/s12870-021-03001-w)
Supplement: Supplementary file 1 — Additional file 1: Table S1. Summary of RNA-seq data and mapped reads for mRNA. [file 12870_2021_3001_MOESM1_ESM.docx]

**Table S1.** Summary of RNA-seq data and mapped reads for mRNA.

| Sample name | Raw reads | Clean reads | Q20(%) | Q30(%) | GC content (%) |
| --- | --- | --- | --- | --- | --- |
| GRS1 | 119384002 | 115611276 | 97.97 | 93.95 | 44.69 |
| GRS2 | 102688854 | 100117026 | 97.96 | 94 | 44.61 |
| GRS3 | 120007214 | 116773710 | 97.98 | 93.98 | 44.4 |
| HG1 | 102527176 | 99573366 | 97.69 | 93.26 | 42.4 |
| HG2 | 118450346 | 114392322 | 97.82 | 93.62 | 43.4 |
| HG3 | 106271572 | 103160468 | 97.24 | 92.29 | 43.18 |
