# Supplementary material for: Metabolome and transcriptome analysis reveals the molecular profiles underlying the ginseng response to rusty root symptoms
Source: BMC Plant Biol. 2021 May 13;21:215. doi: 10.1186/s12870-021-03001-w (PMC8117609; doi:10.1186/s12870-021-03001-w)
Supplement: Supplementary file 2 — Additional file 2: Table S2. Gene Ontology results of all the enriched terms for the upregulated mRNAs in GRS tissues. [file 12870_2021_3001_MOESM2_ESM.docx]

**Table S2.** Gene Ontology results of all the enriched terms for the mRNAs upregulated in GRS tissues.

| **GO_ID** | **Description** | **Term_type** | **pValue** | **qValue** | **DEG_item** | **DEG_list** | **Bg_item** |
| --- | --- | --- | --- | --- | --- | --- | --- |
| GO:0055114 | oxidation-reduction process | biological_process | 1.90E-18 | 9.06E-15 | 158 | 811 | 4403 |
| GO:0016491 | oxidoreductase activity | molecular_function | 1.29E-17 | 3.09E-14 | 160 | 811 | 4597 |
| GO:0005975 | carbohydrate metabolic process | biological_process | 3.65E-13 | 4.36E-10 | 93 | 811 | 2312 |
| GO:0008150 | biological_process | biological_process | 3.65E-13 | 4.36E-10 | 669 | 811 | 33004 |
| GO:0004553 | hydrolase activity, hydrolyzing O-glycosyl compounds | molecular_function | 6.25E-12 | 5.97E-09 | 53 | 811 | 1038 |
| GO:0044710 | single-organism metabolic process | biological_process | 1.18E-11 | 9.37E-09 | 244 | 811 | 9259 |
| GO:0003824 | catalytic activity | molecular_function | 1.78E-11 | 1.22E-08 | 492 | 811 | 22366 |
| GO:0008152 | metabolic process | biological_process | 3.25E-11 | 1.94E-08 | 529 | 811 | 24856 |
| GO:0016798 | hydrolase activity, acting on glycosyl bonds | molecular_function | 1.15E-10 | 6.09E-08 | 53 | 811 | 1122 |
| GO:0020037 | heme binding | molecular_function | 1.19E-09 | 5.70E-07 | 41 | 811 | 846 |
| GO:0046906 | tetrapyrrole binding | molecular_function | 2.80E-09 | 1.22E-06 | 41 | 811 | 870 |
| GO:0004601 | peroxidase activity | molecular_function | 6.69E-08 | 2.66E-05 | 19 | 811 | 240 |
| GO:0016684 | oxidoreductase activity, acting on peroxide as acceptor | molecular_function | 1.87E-07 | 6.89E-05 | 19 | 811 | 256 |
| GO:0006979 | response to oxidative stress | biological_process | 2.41E-07 | 8.22E-05 | 18 | 811 | 238 |
| GO:0016705 | oxidoreductase activity, acting on paired donors, with incorporation or reduction of molecular oxygen | molecular_function | 3.24E-07 | 0.000103 | 37 | 811 | 877 |
| GO:0006694 | steroid biosynthetic process | biological_process | 3.56E-07 | 0.000106 | 18 | 811 | 234 |
| GO:0071554 | cell wall organization or biogenesis | biological_process | 6.23E-07 | 0.000169 | 18 | 811 | 263 |
| GO:0008202 | steroid metabolic process | biological_process | 6.35E-07 | 0.000169 | 18 | 811 | 243 |
| GO:0016717 | oxidoreductase activity, acting on paired donors, with oxidation of a pair of donors resulting in the reduction of molecular oxygen to two molecules of water | molecular_function | 1.68E-06 | 0.000423 | 10 | 811 | 92 |
| GO:0003854 | 3-beta-hydroxy-delta5-steroid dehydrogenase activity | molecular_function | 2.02E-06 | 0.000439 | 16 | 811 | 212 |
| GO:0016229 | steroid dehydrogenase activity | molecular_function | 2.02E-06 | 0.000439 | 16 | 811 | 212 |
| GO:0033764 | steroid dehydrogenase activity, acting on the CH-OH group of donors, NAD or NADP as acceptor | molecular_function | 2.02E-06 | 0.000439 | 16 | 811 | 212 |
| GO:0048037 | cofactor binding | molecular_function | 3.23E-06 | 0.000671 | 56 | 811 | 1632 |
| GO:0044699 | single-organism process | biological_process | 4.60E-06 | 0.000916 | 363 | 811 | 16906 |
| GO:0043565 | sequence-specific DNA binding | molecular_function | 6.16E-06 | 0.001179 | 37 | 811 | 951 |
| GO:0016209 | antioxidant activity | molecular_function | 6.69E-06 | 0.00123 | 21 | 811 | 383 |
| GO:0005506 | iron ion binding | molecular_function | 8.32E-06 | 0.001474 | 31 | 811 | 763 |
| GO:0051382 | kinetochore assembly | biological_process | 1.16E-05 | 0.001908 | 8 | 811 | 63 |
| GO:0051383 | kinetochore organization | biological_process | 1.16E-05 | 0.001908 | 8 | 811 | 63 |
| GO:0016051 | carbohydrate biosynthetic process | biological_process | 1.50E-05 | 0.002394 | 23 | 811 | 457 |
| GO:0004097 | catechol oxidase activity | molecular_function | 1.60E-05 | 0.002467 | 4 | 811 | 11 |
| GO:0005618 | cell wall | cellular_component | 1.85E-05 | 0.002771 | 14 | 811 | 220 |
| GO:0030599 | pectinesterase activity | molecular_function | 2.11E-05 | 0.00296 | 11 | 811 | 140 |
| GO:0042545 | cell wall modification | biological_process | 2.11E-05 | 0.00296 | 11 | 811 | 140 |
| GO:0071555 | cell wall organization | biological_process | 3.12E-05 | 0.004108 | 13 | 811 | 199 |
| GO:0001071 | nucleic acid binding transcription factor activity | molecular_function | 3.18E-05 | 0.004108 | 55 | 811 | 1808 |
| GO:0003700 | transcription factor activity, sequence-specific DNA binding | molecular_function | 3.18E-05 | 0.004108 | 55 | 811 | 1808 |
| GO:0044262 | cellular carbohydrate metabolic process | biological_process | 3.67E-05 | 0.004623 | 22 | 811 | 457 |
| GO:0006026 | aminoglycan catabolic process | biological_process | 4.57E-05 | 0.005608 | 6 | 811 | 39 |
| GO:0045229 | external encapsulating structure organization | biological_process | 4.96E-05 | 0.005923 | 13 | 811 | 207 |
| GO:0004568 | chitinase activity | molecular_function | 5.97E-05 | 0.006956 | 5 | 811 | 25 |
| GO:0016998 | cell wall macromolecule catabolic process | biological_process | 7.98E-05 | 0.009083 | 5 | 811 | 27 |
| GO:0034508 | centromere complex assembly | biological_process | 0.000114 | 0.012651 | 8 | 811 | 85 |
| GO:0050662 | coenzyme binding | molecular_function | 0.000127 | 0.013843 | 44 | 811 | 1360 |
| GO:0030312 | external encapsulating structure | cellular_component | 0.000139 | 0.014777 | 15 | 811 | 293 |
| GO:0009341 | beta-galactosidase complex | cellular_component | 0.000202 | 0.020803 | 6 | 811 | 43 |
| GO:0015925 | galactosidase activity | molecular_function | 0.000208 | 0.020803 | 7 | 811 | 62 |
| GO:0044711 | single-organism biosynthetic process | biological_process | 0.000209 | 0.020803 | 71 | 811 | 2541 |
| GO:0016901 | oxidoreductase activity, acting on the CH-OH group of donors, quinone or similar compound as acceptor | molecular_function | 0.000214 | 0.020857 | 3 | 811 | 7 |
| GO:0016614 | oxidoreductase activity, acting on CH-OH group of donors | molecular_function | 0.000225 | 0.021524 | 28 | 811 | 737 |
| GO:0006032 | chitin catabolic process | biological_process | 0.000241 | 0.021756 | 5 | 811 | 33 |
| GO:0046348 | amino sugar catabolic process | biological_process | 0.000241 | 0.021756 | 5 | 811 | 33 |
| GO:1901072 | glucosamine-containing compound catabolic process | biological_process | 0.000241 | 0.021756 | 5 | 811 | 33 |
| GO:0016682 | oxidoreductase activity, acting on diphenols and related substances as donors, oxygen as acceptor | molecular_function | 0.00027 | 0.023871 | 5 | 811 | 33 |
| GO:0034637 | cellular carbohydrate biosynthetic process | biological_process | 0.000286 | 0.024901 | 16 | 811 | 318 |
| GO:0006082 | organic acid metabolic process | biological_process | 0.000374 | 0.031944 | 49 | 811 | 1602 |
| GO:0004565 | beta-galactosidase activity | molecular_function | 0.000413 | 0.034615 | 6 | 811 | 50 |
| GO:0044723 | single-organism carbohydrate metabolic process | biological_process | 0.000422 | 0.03477 | 37 | 811 | 1111 |
| GO:1901605 | alpha-amino acid metabolic process | biological_process | 0.000482 | 0.039067 | 20 | 811 | 472 |
| GO:0043436 | oxoacid metabolic process | biological_process | 0.000562 | 0.044759 | 48 | 811 | 1589 |
| GO:0006952 | defense response | biological_process | 0.000591 | 0.046311 | 22 | 811 | 586 |
| GO:0006541 | glutamine metabolic process | biological_process | 0.000608 | 0.046904 | 7 | 811 | 78 |
| GO:0016160 | amylase activity | molecular_function | 0.000635 | 0.047907 | 5 | 811 | 38 |
| GO:0005507 | copper ion binding | molecular_function | 0.000641 | 0.047907 | 12 | 811 | 222 |
| GO:0005976 | polysaccharide metabolic process | biological_process | 0.000664 | 0.048803 | 16 | 811 | 351 |
