# Supplementary material for: Metabolome and transcriptome analysis reveals the molecular profiles underlying the ginseng response to rusty root symptoms
Source: BMC Plant Biol. 2021 May 13;21:215. doi: 10.1186/s12870-021-03001-w (PMC8117609; doi:10.1186/s12870-021-03001-w)
Supplement: Supplementary file 3 — Additional file 3: Table S3. Gene Ontology results of all the enriched terms for the downregulated mRNAs in GRS tissues. [file 12870_2021_3001_MOESM3_ESM.docx]

**Table S3.** Gene Ontology results of all the enriched terms for the mRNAs downregulated in GRS tissues.

| **GO_ID** | **Description** | **Term_type** | **pValue** | **qValue** | **DEG_item** | **DEG_list** | **Bg_item** |
| --- | --- | --- | --- | --- | --- | --- | --- |
| GO:0016491 | oxidoreductase activity | molecular_function | 9.57E-21 | 4.57E-17 | 158 | 740 | 4597 |
| GO:0003824 | catalytic activity | molecular_function | 5.52E-20 | 1.32E-16 | 485 | 740 | 22366 |
| GO:0055114 | oxidation-reduction process | biological_process | 1.35E-19 | 2.15E-16 | 151 | 740 | 4403 |
| GO:0044710 | single-organism metabolic process | biological_process | 2.77E-14 | 3.31E-11 | 236 | 740 | 9259 |
| GO:0016705 | oxidoreductase activity, acting on paired donors, with incorporation or reduction of molecular oxygen | molecular_function | 3.92E-14 | 3.75E-11 | 48 | 740 | 877 |
| GO:0020037 | heme binding | molecular_function | 2.58E-12 | 2.05E-09 | 44 | 740 | 846 |
| GO:0046906 | tetrapyrrole binding | molecular_function | 6.78E-12 | 4.63E-09 | 44 | 740 | 870 |
| GO:0006720 | isoprenoid metabolic process | biological_process | 1.43E-10 | 7.02E-08 | 21 | 740 | 220 |
| GO:0008299 | isoprenoid biosynthetic process | biological_process | 1.43E-10 | 7.02E-08 | 21 | 740 | 220 |
| GO:0044255 | cellular lipid metabolic process | biological_process | 1.47E-10 | 7.02E-08 | 47 | 740 | 1002 |
| GO:0006629 | lipid metabolic process | biological_process | 1.82E-10 | 7.93E-08 | 66 | 740 | 1741 |
| GO:0033897 | ribonuclease T2 activity | molecular_function | 4.95E-10 | 1.97E-07 | 10 | 740 | 39 |
| GO:0006721 | terpenoid metabolic process | biological_process | 2.17E-09 | 7.40E-07 | 17 | 740 | 161 |
| GO:0016114 | terpenoid biosynthetic process | biological_process | 2.17E-09 | 7.40E-07 | 17 | 740 | 161 |
| GO:0016798 | hydrolase activity, acting on glycosyl bonds | molecular_function | 2.81E-09 | 8.97E-07 | 47 | 740 | 1122 |
| GO:0005506 | iron ion binding | molecular_function | 3.89E-09 | 1.16E-06 | 36 | 740 | 763 |
| GO:0044699 | single-organism process | biological_process | 1.34E-08 | 3.68E-06 | 346 | 740 | 16906 |
| GO:0016892 | endoribonuclease activity, producing 3'-phosphomonoesters | molecular_function | 1.39E-08 | 3.68E-06 | 10 | 740 | 54 |
| GO:0048037 | cofactor binding | molecular_function | 1.86E-08 | 4.69E-06 | 58 | 740 | 1632 |
| GO:0050662 | coenzyme binding | molecular_function | 2.29E-08 | 5.31E-06 | 51 | 740 | 1360 |
| GO:0016894 | endonuclease activity, active with either ribo- or deoxyribonucleic acids and producing 3'-phosphomonoesters | molecular_function | 2.33E-08 | 5.31E-06 | 10 | 740 | 57 |
| GO:0008152 | metabolic process | biological_process | 2.70E-08 | 5.86E-06 | 471 | 740 | 24856 |
| GO:0050660 | flavin adenine dinucleotide binding | molecular_function | 1.21E-07 | 2.51E-05 | 26 | 740 | 497 |
| GO:0003857 | 3-hydroxyacyl-CoA dehydrogenase activity | molecular_function | 1.60E-07 | 3.19E-05 | 12 | 740 | 106 |
| GO:0004553 | hydrolase activity, hydrolyzing O-glycosyl compounds | molecular_function | 1.80E-07 | 3.45E-05 | 41 | 740 | 1038 |
| GO:0042440 | pigment metabolic process | biological_process | 2.48E-07 | 4.56E-05 | 19 | 740 | 285 |
| GO:0008150 | biological_process | biological_process | 7.23E-07 | 0.000128 | 586 | 740 | 33004 |
| GO:0016108 | tetraterpenoid metabolic process | biological_process | 1.76E-06 | 0.000271 | 11 | 740 | 108 |
| GO:0016109 | tetraterpenoid biosynthetic process | biological_process | 1.76E-06 | 0.000271 | 11 | 740 | 108 |
| GO:0016116 | carotenoid metabolic process | biological_process | 1.76E-06 | 0.000271 | 11 | 740 | 108 |
| GO:0016117 | carotenoid biosynthetic process | biological_process | 1.76E-06 | 0.000271 | 11 | 740 | 108 |
| GO:0004601 | peroxidase activity | molecular_function | 2.11E-06 | 0.000316 | 16 | 740 | 240 |
| GO:0032787 | monocarboxylic acid metabolic process | biological_process | 2.63E-06 | 0.00038 | 27 | 740 | 599 |
| GO:0004616 | phosphogluconate dehydrogenase (decarboxylating) activity | molecular_function | 4.37E-06 | 0.000614 | 10 | 740 | 97 |
| GO:0016684 | oxidoreductase activity, acting on peroxide as acceptor | molecular_function | 4.89E-06 | 0.000668 | 16 | 740 | 256 |
| GO:0005975 | carbohydrate metabolic process | biological_process | 5.18E-06 | 0.000687 | 67 | 740 | 2312 |
| GO:0016209 | antioxidant activity | molecular_function | 5.75E-06 | 0.000743 | 20 | 740 | 383 |
| GO:0008610 | lipid biosynthetic process | biological_process | 6.88E-06 | 0.000865 | 34 | 740 | 892 |
| GO:0046148 | pigment biosynthetic process | biological_process | 7.73E-06 | 0.000948 | 14 | 740 | 200 |
| GO:0055085 | transmembrane transport | biological_process | 8.84E-06 | 0.001057 | 64 | 740 | 2213 |
| GO:0006631 | fatty acid metabolic process | biological_process | 9.28E-06 | 0.001082 | 18 | 740 | 331 |
| GO:0016762 | xyloglucan:xyloglucosyl transferase activity | molecular_function | 2.09E-05 | 0.002325 | 7 | 740 | 57 |
| GO:0048046 | apoplast | cellular_component | 2.09E-05 | 0.002325 | 7 | 740 | 57 |
| GO:0016788 | hydrolase activity, acting on ester bonds | molecular_function | 2.16E-05 | 0.002344 | 58 | 740 | 1993 |
| GO:0006979 | response to oxidative stress | biological_process | 3.25E-05 | 0.003453 | 14 | 740 | 238 |
| GO:0006073 | cellular glucan metabolic process | biological_process | 8.25E-05 | 0.008391 | 14 | 740 | 256 |
| GO:0044042 | glucan metabolic process | biological_process | 8.25E-05 | 0.008391 | 14 | 740 | 256 |
| GO:0016616 | oxidoreductase activity, acting on the CH-OH group of donors, NAD or NADP as acceptor | molecular_function | 8.46E-05 | 0.008426 | 26 | 740 | 684 |
| GO:0044711 | single-organism biosynthetic process | biological_process | 0.000101 | 0.009837 | 67 | 740 | 2541 |
| GO:0016614 | oxidoreductase activity, acting on CH-OH group of donors | molecular_function | 0.00011 | 0.010534 | 27 | 740 | 737 |
| GO:0016746 | transferase activity, transferring acyl groups | molecular_function | 0.000129 | 0.012088 | 29 | 740 | 860 |
| GO:0016787 | hydrolase activity | molecular_function | 0.000133 | 0.012201 | 177 | 740 | 8339 |
| GO:0005618 | cell wall | cellular_component | 0.000153 | 0.013757 | 12 | 740 | 220 |
| GO:0071949 | FAD binding | molecular_function | 0.000177 | 0.015647 | 11 | 740 | 175 |
| GO:1901565 | organonitrogen compound catabolic process | biological_process | 0.000267 | 0.023236 | 11 | 740 | 193 |
| GO:1901136 | carbohydrate derivative catabolic process | biological_process | 0.000305 | 0.026042 | 8 | 740 | 103 |
| GO:0016717 | oxidoreductase activity, acting on paired donors, with oxidation of a pair of donors resulting in the reduction of molecular oxygen to two molecules of water | molecular_function | 0.000394 | 0.03307 | 7 | 740 | 92 |
| GO:0044264 | cellular polysaccharide metabolic process | biological_process | 0.000405 | 0.033419 | 14 | 740 | 297 |
| GO:0004521 | endoribonuclease activity | molecular_function | 0.000484 | 0.039238 | 11 | 740 | 199 |
| GO:0004568 | chitinase activity | molecular_function | 0.000599 | 0.047749 | 4 | 740 | 25 |
