# Supplementary material for: Metabolome and transcriptome analysis reveals the molecular profiles underlying the ginseng response to rusty root symptoms
Source: BMC Plant Biol. 2021 May 13;21:215. doi: 10.1186/s12870-021-03001-w (PMC8117609; doi:10.1186/s12870-021-03001-w)
Supplement: Supplementary file 4 — Additional file 4: Table S4. All pathways associated with DEGs based on KEGG enrichment analysis. [file 12870_2021_3001_MOESM4_ESM.docx]

**Table S4.** All pathways associated with DEGs based on KEGG enrichment analysis.

| **Term** | **ID** | **Input number** | **Background number** | | **pValue** |
| --- | --- | --- | --- | --- | --- |
| Biosynthesis of secondary metabolites | ath01110 | 656 | 995 | 0.004803212 | |
| Phenylpropanoid biosynthesis | ath00940 | 123 | 154 | 0.005133023 | |
| Metabolic pathways | ath01100 | 1173 | 1861 | 0.009174112 | |
| Stilbenoid, diarylheptanoid and gingerol biosynthesis | ath00945 | 58 | 64 | 0.009359597 | |
| Cysteine and methionine metabolism | ath00270 | 79 | 99 | 0.021700816 | |
| Fatty acid biosynthesis | ath00061 | 37 | 40 | 0.027658597 | |
| Tyrosine metabolism | ath00350 | 36 | 40 | 0.03737812 | |
| beta-Alanine metabolism | ath00410 | 35 | 40 | 0.049911444 | |
| Sesquiterpenoid and triterpenoid biosynthesis | ath00909 | 22 | 23 | 0.064406363 | |
| Valine, leucine and isoleucine degradation | ath00280 | 37 | 45 | 0.07393252 | |
| Alanine, aspartate and glutamate metabolism | ath00250 | 39 | 48 | 0.074771629 | |
| Glycerolipid metabolism | ath00561 | 41 | 52 | 0.088270048 | |
| Sphingolipid metabolism | ath00600 | 23 | 26 | 0.093200071 | |
| Ether lipid metabolism | ath00565 | 23 | 26 | 0.093200071 | |
| Ubiquinone and other terpenoid-quinone biosynthesis | ath00130 | 27 | 32 | 0.098134073 | |
| Peroxisome | ath04146 | 59 | 81 | 0.109188932 | |
| Phenylalanine metabolism | ath00360 | 80 | 114 | 0.111556724 | |
| Arachidonic acid metabolism | ath00590 | 16 | 17 | 0.113148316 | |
| Glycine, serine and threonine metabolism | ath00260 | 51 | 69 | 0.113785743 | |
| Photosynthesis | ath00195 | 56 | 77 | 0.117719759 | |
| Isoquinoline alkaloid biosynthesis | ath00950 | 20 | 23 | 0.122330801 | |
| Pyruvate metabolism | ath00620 | 59 | 83 | 0.136585957 | |
| Glyoxylate and dicarboxylate metabolism | ath00630 | 46 | 63 | 0.140590271 | |
| Limonene and pinene degradation | ath00903 | 46 | 63 | 0.140590271 | |
| Selenocompound metabolism | ath00450 | 16 | 18 | 0.143391531 | |
| Pentose and glucuronate interconversions | ath00040 | 57 | 81 | 0.154151769 | |
| Terpenoid backbone biosynthesis | ath00900 | 42 | 58 | 0.162837399 | |
| Glycerophospholipid metabolism | ath00564 | 57 | 83 | 0.188379493 | |
| Amino sugar and nucleotide sugar metabolism | ath00520 | 81 | 122 | 0.195626326 | |
| Fructose and mannose metabolism | ath00051 | 37 | 52 | 0.202288604 | |
| Taurine and hypotaurine metabolism | ath00430 | 12 | 14 | 0.213336425 | |
| Glycosaminoglycan degradation | ath00531 | 7 | 7 | 0.224096646 | |
| Starch and sucrose metabolism | ath00500 | 120 | 188 | 0.228141514 | |
| Circadian rhythm - plant | ath04712 | 26 | 36 | 0.236592915 | |
| Glycolysis / Gluconeogenesis | ath00010 | 73 | 112 | 0.245842821 | |
| Carbon metabolism | ath01200 | 152 | 243 | 0.254452802 | |
| Glycosphingolipid biosynthesis - globo series | ath00603 | 8 | 9 | 0.261129698 | |
| DNA replication | ath03030 | 34 | 50 | 0.275064144 | |
| Galactose metabolism | ath00052 | 37 | 55 | 0.279666846 | |
| One carbon pool by folate | ath00670 | 15 | 20 | 0.280656289 | |
| Butanoate metabolism | ath00650 | 13 | 17 | 0.285525771 | |
| Nicotinate and nicotinamide metabolism | ath00760 | 11 | 14 | 0.289860294 | |
| Thiamine metabolism | ath00730 | 9 | 11 | 0.293077827 | |
| Phenylalanine, tyrosine and tryptophan biosynthesis | ath00400 | 37 | 57 | 0.336736359 | |
| Riboflavin metabolism | ath00740 | 7 | 9 | 0.367401058 | |
| Biosynthesis of amino acids | ath01230 | 154 | 255 | 0.373013779 | |
| Nitrogen metabolism | ath00910 | 27 | 42 | 0.385537304 | |
| ABC transporters | ath02010 | 16 | 24 | 0.392761647 | |
| Steroid biosynthesis | ath00100 | 22 | 34 | 0.396549703 | |
| Pentose phosphate pathway | ath00030 | 34 | 54 | 0.397156083 | |
| Lysine biosynthesis | ath00300 | 11 | 16 | 0.403639388 | |
| Sulfur metabolism | ath00920 | 26 | 41 | 0.409613621 | |
| Plant-pathogen interaction | ath04626 | 96 | 164 | 0.504011645 | |
| Cutin, suberine and wax biosynthesis | ath00073 | 14 | 23 | 0.508557495 | |
| Ascorbate and aldarate metabolism | ath00053 | 24 | 41 | 0.537058991 | |
| Photosynthesis - antenna proteins | ath00196 | 13 | 22 | 0.545709668 | |
| Propanoate metabolism | ath00640 | 13 | 22 | 0.545709668 | |
| Tropane, piperidine and pyridine alkaloid biosynthesis | ath00960 | 21 | 36 | 0.545865739 | |
| Arginine and proline metabolism | ath00330 | 43 | 75 | 0.564981414 | |
| Carbon fixation in photosynthetic organisms | ath00710 | 39 | 69 | 0.593160199 | |
| Diterpenoid biosynthesis | ath00904 | 10 | 18 | 0.616395379 | |
| Histidine metabolism | ath00340 | 10 | 18 | 0.616395379 | |
| Homologous recombination | ath03440 | 31 | 56 | 0.627221529 | |
| Citrate cycle (TCA cycle) | ath00020 | 34 | 62 | 0.646217612 | |
| Valine, leucine and isoleucine biosynthesis | ath00290 | 12 | 23 | 0.680249187 | |
| Lysine degradation | ath00310 | 13 | 25 | 0.686035272 | |
| Glutathione metabolism | ath00480 | 50 | 93 | 0.702919577 | |
| Folate biosynthesis | ath00790 | 11 | 22 | 0.71894689 | |
| 2-Oxocarboxylic acid metabolism | ath01210 | 39 | 74 | 0.723366601 | |
| Mismatch repair | ath03430 | 19 | 39 | 0.778412271 | |
| Phagosome | ath04145 | 43 | 86 | 0.817136616 | |
| C5-Branched dibasic acid metabolism | ath00660 | 4 | 10 | 0.818202148 | |
| Pyrimidine metabolism | ath00240 | 59 | 116 | 0.821875082 | |
| Cyanoamino acid metabolism | ath00460 | 29 | 60 | 0.824184877 | |
| Carotenoid biosynthesis | ath00906 | 13 | 29 | 0.826996195 | |
| Oxidative phosphorylation | ath00190 | 83 | 162 | 0.845012184 | |
| Vitamin B6 metabolism | ath00750 | 5 | 13 | 0.850572225 | |
| Biotin metabolism | ath00780 | 5 | 14 | 0.884134284 | |
| Purine metabolism | ath00230 | 78 | 157 | 0.888651595 | |
| Proteasome | ath03050 | 26 | 58 | 0.890508539 | |
| Phosphatidylinositol signaling system | ath04070 | 30 | 66 | 0.892173974 | |
| Inositol phosphate metabolism | ath00562 | 29 | 65 | 0.904889145 | |
| RNA polymerase | ath03020 | 19 | 45 | 0.906302916 | |
| Fatty acid elongation | ath00062 | 12 | 31 | 0.916475175 | |
| Tryptophan metabolism | ath00380 | 18 | 45 | 0.93338737 | |
| Pantothenate and CoA biosynthesis | ath00770 | 10 | 28 | 0.937318283 | |
| Plant hormone signal transduction | ath04075 | 135 | 271 | 0.93950348 | |
| Zeatin biosynthesis | ath00908 | 7 | 23 | 0.961251994 | |
| Glucosinolate biosynthesis | ath00966 | 5 | 19 | 0.971419454 | |
| Regulation of autophagy | ath04140 | 8 | 27 | 0.973984138 | |
| Non-homologous end-joining | ath03450 | 1 | 8 | 0.983881738 | |
| Porphyrin and chlorophyll metabolism | ath00860 | 14 | 45 | 0.988781284 | |
| Ubiquitin mediated proteolysis | ath04120 | 62 | 149 | 0.990505846 | |
| Endocytosis | ath04144 | 46 | 117 | 0.991764194 | |
| Sulfur relay system | ath04122 | 2 | 14 | 0.993303338 | |
| Nucleotide excision repair | ath03420 | 23 | 69 | 0.994166254 | |
| Base excision repair | ath03410 | 12 | 43 | 0.994378705 | |
| Protein export | ath03060 | 16 | 53 | 0.99461907 | |
| Protein processing in endoplasmic reticulum | ath04141 | 88 | 220 | 0.999116599 | |
| RNA transport | ath03013 | 60 | 169 | 0.999761293 | |
| Ribosome biogenesis in eukaryotes | ath03008 | 30 | 101 | 0.999780711 | |
| SNARE interactions in vesicular transport | ath04130 | 9 | 48 | 0.999871753 | |
| Glycosylphosphatidylinositol (GPI)-anchor biosynthesis | ath00563 | 2 | 24 | 0.999893782 | |
| Spliceosome | ath03040 | 62 | 192 | 0.999991858 | |
| RNA degradation | ath03018 | 28 | 111 | 0.999993595 | |
| N-Glycan biosynthesis | ath00510 | 5 | 44 | 0.999995171 | |
| Basal transcription factors | ath03022 | 8 | 55 | 0.999995687 | |
| mRNA surveillance pathway | ath03015 | 24 | 114 | 0.999999841 | |
| Aminoacyl-tRNA biosynthesis | ath00970 | 17 | 115 | 1 | |
| Ribosome | ath03010 | 67 | 360 | 1 | |
