# Supplementary material for: Metabolome and transcriptome analysis reveals the molecular profiles underlying the ginseng response to rusty root symptoms
Source: BMC Plant Biol. 2021 May 13;21:215. doi: 10.1186/s12870-021-03001-w (PMC8117609; doi:10.1186/s12870-021-03001-w)
Supplement: Supplementary file 7 — Additional file 7: Table S7. Primer sequences of the genes for qPCR verification. [file 12870_2021_3001_MOESM7_ESM.docx]

| Name | Primer | Sequence | Size |
| --- | --- | --- | --- |
| ACTIN | Forward | 5‘-CTTGCACCAAGCAGCATGAA-3’ | 67bp |
|  | Reverse | 5‘-CCGATCCAGACACTGTACTTCCTT-3’ |  |
| Pg_S1410.4 | Forward | 5‘-GCCGTCCGCTCTAAGAAACTACC-3’ | 105bp |
|  | Reverse | 5‘-TCTCATCTCAGCACCTTCCTCCTC-3’ |  |
| Pg_S1825.18 | Forward | 5‘-CGGCAAGATGGTGGCGGAAC-3’ | 104bp |
|  | Reverse | 5‘-GCTGTAACGGCGGTCCAAGAAC-3’ |  |
| Pg_S2641.9 | Forward | 5‘-TGGAACTGCCACCTTCACAATCAC-3’ | 87bp |
|  | Reverse | 5‘-GCATGGACAACAACAGCCCTACC-3’ |  |
| Pg_S7602.1 | Forward | 5‘-CACTGCACTTCCGGGTCATCTTG-3’ | 144bp |
|  | Reverse | 5‘-GGCTTGAGTTCTCTGTCACGATCC-3’ |  |
| Pg_S4621.10 | Forward | 5‘-ATCTTAGGGTTTGCGTGGGAAAGC-3’ | 95bp |
|  | Reverse | 5‘-GCAGAGCCGTGAATGGTGTCC-3’ |  |
| Pg_S1378.3 | Forward | 5‘-TTGTGGACTCTCCCTGCTGTCTAG-3’ | 80bp |
|  | Reverse | 5‘-CGCCTGTGCCTTCATAATCCTCTC-3’ |  |
| Pg_S3064.15 | Forward | 5‘-CCGAGCCCCAAGCCTGTTTATC-3’ | 99bp |
|  | Reverse | 5‘-GGACCGAAGAGCGTTATGTGGATC-3’ |  |
| Pg_S0213.26 | Forward | 5‘-CCTCCTCCTCCTCCACACCTTG-3’ | 80bp |
|  | Reverse | 5‘-GAATCGTGAGCCTGCCCTTGATG-3’ |  |

**Table S7.** Primer sequences of the genes for qPCR verification.
