# Supplementary material for: Metabolome and transcriptome analysis reveals the molecular profiles underlying the ginseng response to rusty root symptoms
Source: BMC Plant Biol. 2021 May 13;21:215. doi: 10.1186/s12870-021-03001-w (PMC8117609; doi:10.1186/s12870-021-03001-w)
Supplement: Supplementary file 8 — Additional file 8: Figure S1. Plant hormone contents and heatmaps of DEGs. [file 12870_2021_3001_MOESM8_ESM.docx]

**Fig. S1.** Plant hormone contents and heatmaps of DEGs. (A) levels of ABA, SA and MeJA in ginseng tissues; (B) DEGs related to ABA signal transduction; (C) DEGs related to JA signal transduction; (D) DEGs related to SA signal transduction. HG: healthy ginseng; GRS: Ginseng rusty root symptom.
