# Supplementary material for: Metabolome and transcriptome analysis reveals the molecular profiles underlying the ginseng response to rusty root symptoms
Source: BMC Plant Biol. 2021 May 13;21:215. doi: 10.1186/s12870-021-03001-w (PMC8117609; doi:10.1186/s12870-021-03001-w)
Supplement: Supplementary file 9 — Additional file 9: Figure S2. The plant hormone signal transduction pathway. [file 12870_2021_3001_MOESM9_ESM.docx]

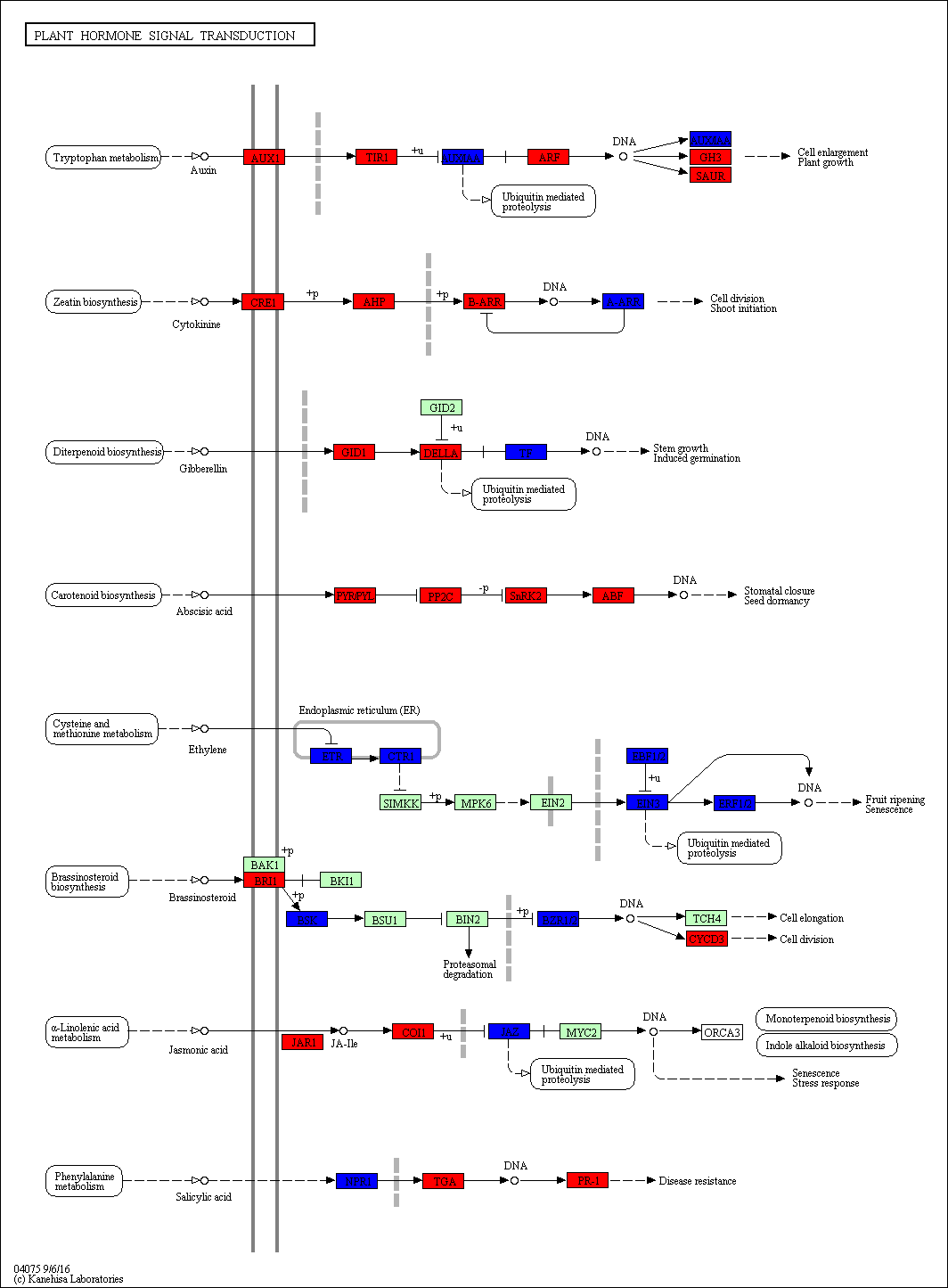


**Fig. S2.** The plant hormone signal transduction pathway. The different color of the frame indicates the different gene transcription in GRS compared with HG (Red frame: up-regulation; blue frame: down-regulation). HG: healthy ginseng; GRS: ginseng rusty root symptoms.
