# Supplementary material for: Metabolome and transcriptome analysis reveals the molecular profiles underlying the ginseng response to rusty root symptoms
Source: BMC Plant Biol. 2021 May 13;21:215. doi: 10.1186/s12870-021-03001-w (PMC8117609; doi:10.1186/s12870-021-03001-w)
Supplement: Supplementary file 10 — Additional file 10: Figure S3. Heatmap of DEGs related to peroxisome synthesis. [file 12870_2021_3001_MOESM10_ESM.docx]

**Fig. S3.** Heatmap of DEGs related to peroxisome synthesis. HG: healthy ginseng; GRS: ginseng rusty root symptoms.
