# Supplementary material for: Metabolome and transcriptome analysis reveals the molecular profiles underlying the ginseng response to rusty root symptoms
Source: BMC Plant Biol. 2021 May 13;21:215. doi: 10.1186/s12870-021-03001-w (PMC8117609; doi:10.1186/s12870-021-03001-w)
Supplement: Supplementary file 12 — Additional file 12: Figure S5. The plant-pathogen interaction pathway. [file 12870_2021_3001_MOESM12_ESM.docx]

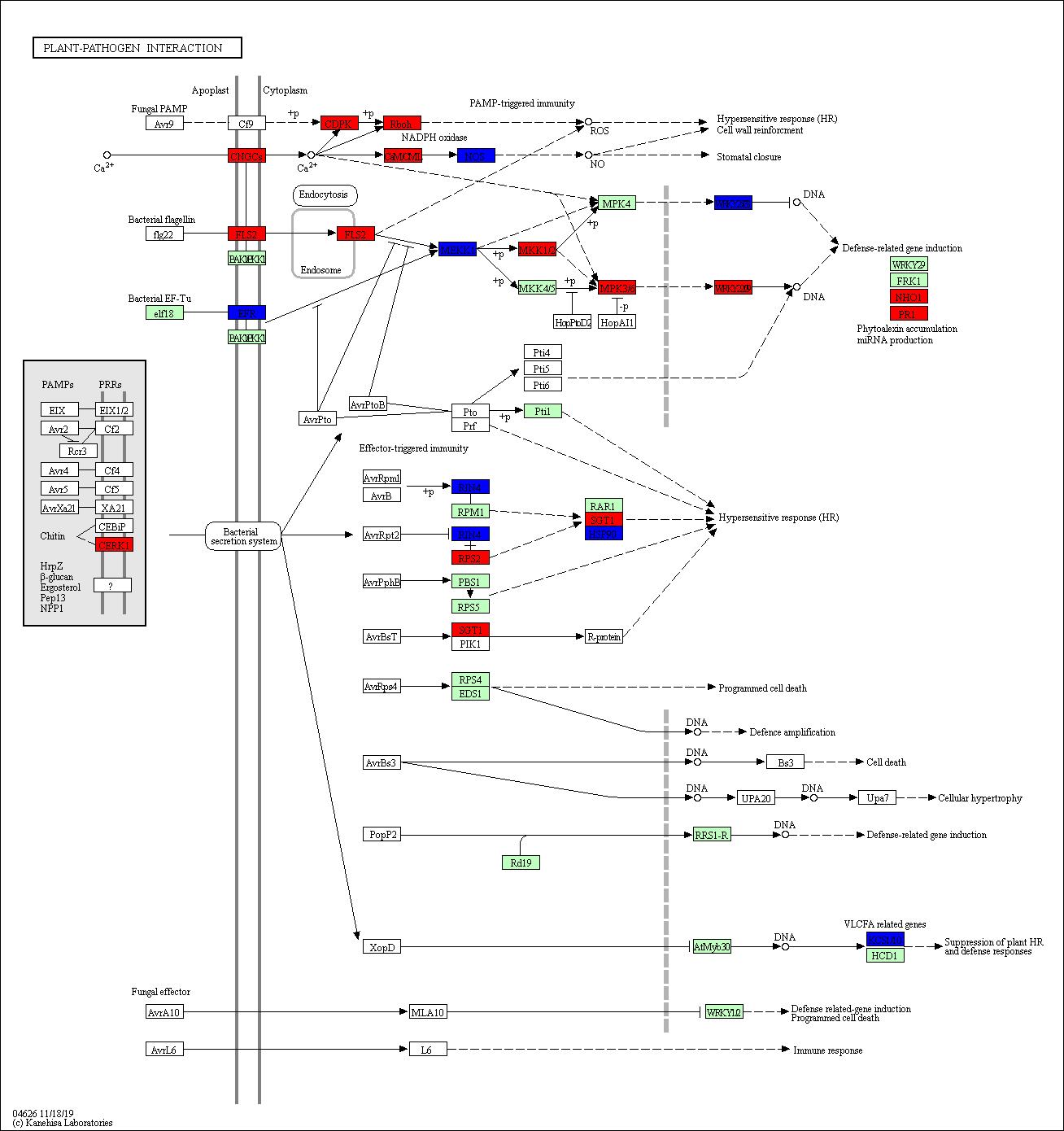


**Figure S5.** The plant-pathogen interaction pathway. The different color of the frame indicates the different gene transcription in GRS compared with HG (Red frame: up-regulation; blue frame: down-regulation). HG: healthy ginseng; GRS: Ginseng rusty root symptom.
