# Supplementary material for: Metabolome and transcriptome analysis reveals the molecular profiles underlying the ginseng response to rusty root symptoms
Source: BMC Plant Biol. 2021 May 13;21:215. doi: 10.1186/s12870-021-03001-w (PMC8117609; doi:10.1186/s12870-021-03001-w)
Supplement: Supplementary file 13 — Additional file 13: Figure S6. Heatmap of DEGs related to plant-pathogen interaction. [file 12870_2021_3001_MOESM13_ESM.docx]

**Fig. S6.** Heatmap of DEGs related to plant-pathogen interaction. HG: healthy ginseng; GRS: Ginseng rusty root symptom.
